# What scans we will read: imaging instrumentation trends in clinical oncology

**DOI:** 10.1186/s40644-020-00312-3

**Published:** 2020-06-09

**Authors:** Thomas Beyer, Luc Bidaut, John Dickson, Marc Kachelriess, Fabian Kiessling, Rainer Leitgeb, Jingfei Ma, Lalith Kumar Shiyam Sundar, Benjamin Theek, Osama Mawlawi

**Affiliations:** 1 QIMP Team, Centre for Medical Physics and Biomedical Engineering, Medical University Vienna, Währinger Gürtel 18-20/4L, 1090 Vienna, Austria; 2 College of Science, University of Lincoln, Lincoln, UK; 3 Institute of Nuclear Medicine, University College London Hospital, London, UK; 4 Division of X-ray imaging and CT, German Cancer Research Center (DKFZ), Im Neuenheimer Feld 280, 69120 Heidelberg, DE Germany; 5 Institute for Experimental Molecular Imaging, University Clinic and Helmholtz Institute for Biomedical Engineering, RWTH Aachen University, Pauwelsstrasse 20, 52074 Aachen, DE Germany; 6 Fraunhofer Institute for Digital Medicine MEVIS, Am Fallturm 1, 28359 Bremen, DE Germany; 7 Centre for Medical Physics and Biomedical Engineering, Medical University Vienna, Vienna, AT Austria; 8 Department of Imaging Physics, The University of Texas MD Anderson Cancer Center, Houston, TX USA

**Keywords:** Oncology imaging, Instrumentation, CT, MRI, Optical, SPECT, US, Sonography, Hybrid imaging, Machine learning

## Abstract

Oncological diseases account for a significant portion of the burden on public healthcare systems with associated costs driven primarily by complex and long-lasting therapies. Through the visualization of patient-specific morphology and functional-molecular pathways, cancerous tissue can be detected and characterized non-invasively, so as to provide referring oncologists with essential information to support therapy management decisions. Following the onset of stand-alone anatomical and functional imaging, we witness a push towards integrating molecular image information through various methods, including anato-metabolic imaging (e.g., PET/CT), advanced MRI, optical or ultrasound imaging.

This perspective paper highlights a number of key technological and methodological advances in imaging instrumentation related to anatomical, functional, molecular medicine and hybrid imaging, that is understood as the hardware-based combination of complementary anatomical and molecular imaging. These include novel detector technologies for ionizing radiation used in CT and nuclear medicine imaging, and novel system developments in MRI and optical as well as opto-acoustic imaging. We will also highlight new data processing methods for improved non-invasive tissue characterization. Following a general introduction to the role of imaging in oncology patient management we introduce imaging methods with well-defined clinical applications and potential for clinical translation. For each modality, we report first on the *status quo* and, then point to perceived technological and methodological advances in a subsequent *status go* section. Considering the breadth and dynamics of these developments, this perspective ends with a critical reflection on where the authors, with the majority of them being imaging experts with a background in physics and engineering, believe imaging methods will be in a few years from now.

Overall, methodological and technological medical imaging advances are geared towards increased image contrast, the derivation of reproducible quantitative parameters, an increase in volume sensitivity and a reduction in overall examination time. To ensure full translation to the clinic, this progress in technologies and instrumentation is complemented by advances in relevant acquisition and image-processing protocols and improved data analysis. To this end, we should accept diagnostic images as “data”, and – through the wider adoption of advanced analysis, including machine learning approaches and a “big data” concept – move to the next stage of non-invasive tumour phenotyping. The scans we will be reading in 10 years from now will likely be composed of highly diverse multi-dimensional data from multiple sources, which mandate the use of advanced and interactive visualization and analysis platforms powered by Artificial Intelligence (AI) for real-time data handling by cross-specialty clinical experts with a domain knowledge that will need to go beyond that of plain imaging.

## Cancer and imaging – an introduction

Cancer describes a wide range of oncological diseases that can affect all levels of a living organism and that have, in common, the risk of becoming systemic. Cancer is the second leading cause of death of people with about 17 million new cases worldwide per year. Just under 10 million people succumb to cancer every year [1], and one in three people is affected by cancer throughout their lifetime. The economic impact of cancer – from diagnosis, treatment, patient work-up and care, to loss of work force and other societal impacts – is thus significant and increasing; in 2010, for example, the worldwide direct and indirect costs were estimated to be 1.6 trillion USD, which amounted to 230 USD per human capita [2]. Typically, overall costs of cancer account for 10%, or more of the gross domestic product (GDP) with marked variations across countries. Given the severe implications of disseminated cancer, early and accurate diagnosis is of the essence.

**Fig. 1:**
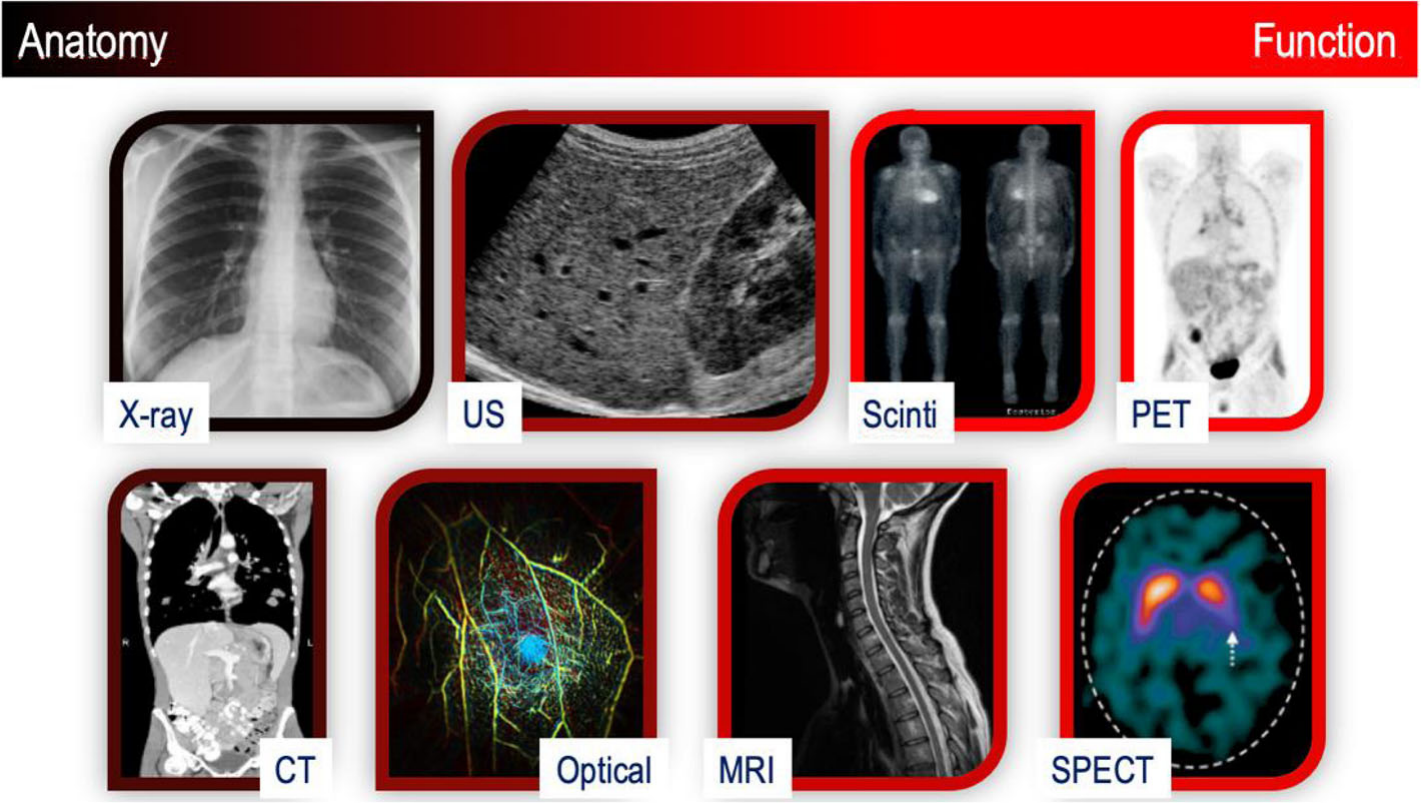
Imaging modalities together with their colour-coded ability to depict anatomical and/or functional information. For example, X-ray imaging is purely anatomical imaging modality, PET imaging provides functional (and molecular) data, while MRI and optical imaging are capable of providing anatomical and functional information depending on the choice of the protocol or mode of operation

Imaging by means of different, and frequently complementary imaging methods provides fundamental data for diagnosing patients, studying diseases, discovering and monitoring new therapies, and improving human health care. As such, the adoption rate of non-invasive, medical imaging has been increasing continuously over the past decades [3]. These increases can be attributed to expanded demand from referring physicians and patients, as well as from technical improvements and wider availability. More specifically, modern biomedical images reveal structural and functional information of subjects in vivo (Fig. 1). At a different scale, biological microscopy images and molecular pathways provide further insight into tissues and living organisms, and into processes and structures of cellular compartments [4]. Given the different types of data generated and scale of information, new ways of integrating and using biomedical information must be found [5]. In this paper, we describe important types of biomedical imaging methods and hypothesize on their short- to mid-term developments with a focus on oncological imaging.

**Fig. 2:**
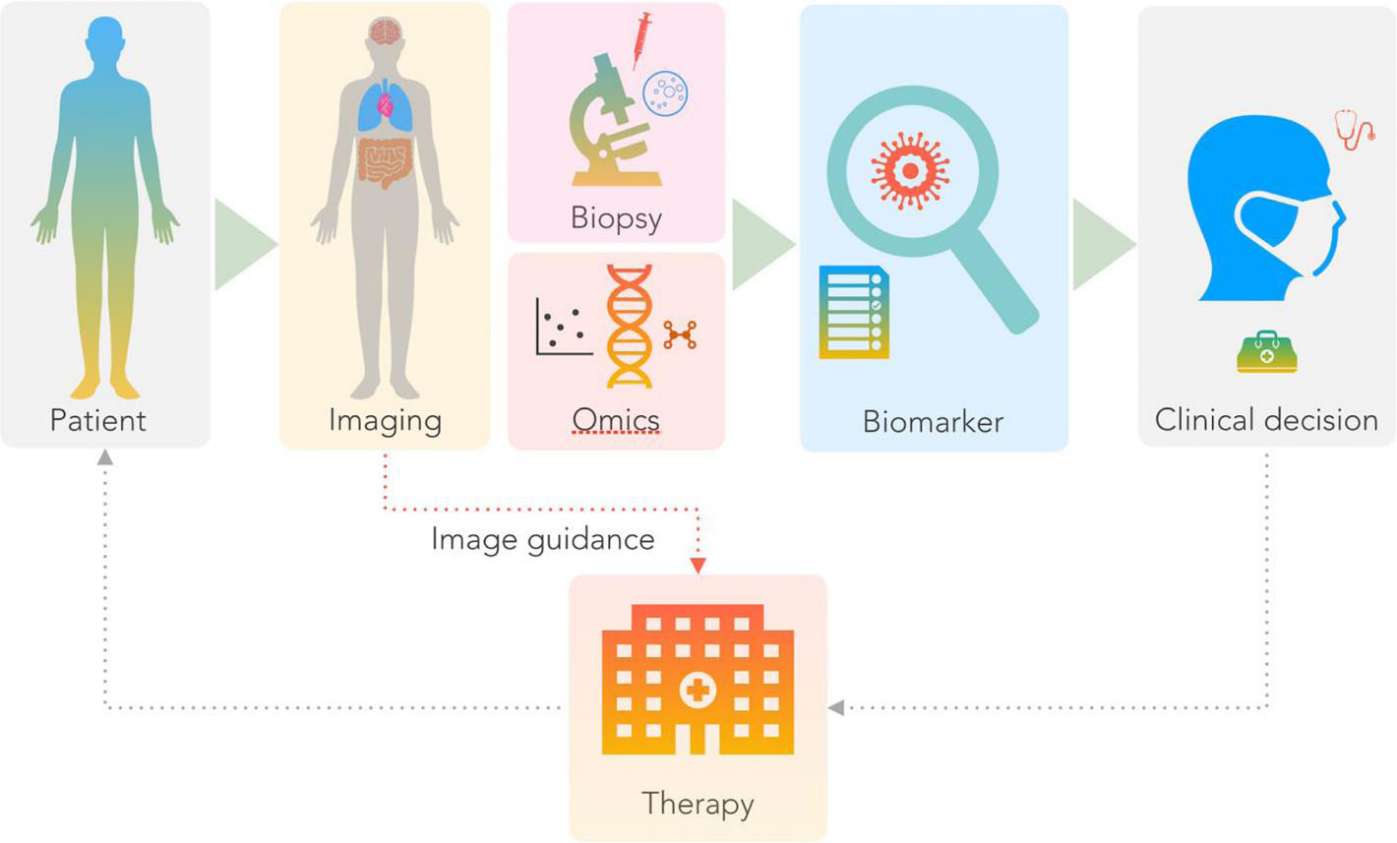
Schematics of a generic management of cancer patients: frequently, a conventional imaging exam (X-ray, CT, and alike) is performed before an additional, combined imaging exam (e.g., PET/CT) is ordered. Imaging may be followed by a biopsy procedure or genomic analysis to further differentiate the disease. A report is created and the oncologist decides on the choice of an appropriate therapy. It is evident that imaging is the gatekeeper to important clinical decision making and therapy deliveries. This cycle can be re-iterated multiple times during patient management and follow-up. The actual implementation of this patient management chart will be affected by the availability of imaging modalities, reimbursement guidelines and other locally variant healthcare regulations

Figure 2 depicts a generic view on the work-up of a patient suspected with cancer: the patient presents with a suspicion of cancer and is referred for an imaging examination. Here, imaging denotes the acquisition of non-invasive visual data of extended ranges or volumes of the subject. Conventional imaging, as indicated in the schematics, typically includes X-ray imaging, Computed Tomography (CT) imaging or Ultrasound (US) or Magnetic Resonance Imaging (MRI), and, thus, yields anatomical information, which can be employed to detect, localize and describe cancerous tissue in-vivo. However, cancer is frequently not detectable from plain anatomical images because of the lack of morphological alterations, but can be identified by virtue of molecular and metabolic perturbations [4].

Nuclear medicine techniques, such as Positron Emission Tomography (PET) or Single Photon Emission Computed Tomography (SPECT), that rely on the tracer principle, have found an important place in the diagnostic management of cancer [6]. The tracer principle describes the ability to label minute amounts of a biomolecule of choice (e.g., glucose) with a radioactive isotope, which enables tracing the labelled biomolecule (and, thus, the pathways of the unlabeled biomolecules) by means of the emitted radiation without disturbing normal tissue function. Likewise, optical imaging methods make use of fluorescent molecular probes and ultrasound of targeted microbubbles to highlight signaling pathways and differential anatomies, respectively [7]. Nuclear medicine imaging can be both highly-sensitive and highly-specific, but yields images of the tracer distribution that are of lower spatial resolution than that from CT or MRI, given the fundamental differences in the detection principles of molecular and anatomical imaging. It is this difference, and the general challenges of localizing focal uptakes of highly-specific tracers that have led to the development of hybrid imaging methods, including the physical combination of PET and CT (PET/CT), SPECT and CT (SPECT/CT), or PET and MRI (PET/MRI) [8].

Imaging is frequently followed by bioptic sampling to further substantiate a diagnosis (Fig. 2). Both imaging and bioptic information are then combined to make a clinical decision on a preferred therapeutic option, many of which come with a serious cost to the healthcare system. An ideal scenario for a cancer patient work-up would include a timely and accurate diagnosis, and a full understanding of the cancerous phenotype while performing a minimal number of tests, which, in turn, should be minimally invasive, and ultimately, lead to the most appropriate choice of a therapy depending on the stage and biological features of the disease (also called “precision medicine”). Ideally, the use of confirmatory bioptic procedures should be limited since they are costly, time consuming, and invasive. However, biopsies are required to determine the genetic cause of a disease and, thus, help adjust subsequent treatment regimens. Should biopsies be avoided in the future, then imaging methods must be as good as deducing the phenotype of the cancerous disease so as to planning the most efficient, and personalized treatment. Here, molecular or hybrid imaging techniques may be better placed as non-invasive tools than other techniques.

Furthermore, the value of image parameters (also called “biomarkers”) is often sub-optimal due to physical and methodological limitations of the diagnostic methods employed: anatomical imaging, for example, may not detect or characterize a lesion because it is not manifested as a morphological alteration even though cancerous tissue is present. Molecular imaging, on the other hand, may miss even hypermetabolic lesions because they are too small to be detected in view of the partial volume effects (PVE) arising from the limited detector resolution [9] or because the lesion resides in an area of the body that is affected by motion (e.g., respiration), which leads to a smearing of the tracer uptake and a resulting contrast that is insufficient to delineate the lesion. Additionally, high uptake in adjacent tissues can diminish contrast and, therefore, sensitivity for lesion detection. The strengths and weaknesses of each imaging modality, with regards to depicting anato-metabolic details or plain lesion detection will play into the adoption of these modalities as standards of care along the cancer management pathway (Fig. 2). Supplementary Figure 1 further details the use of all seven key imaging modalities presented here for diagnosis, staging, restaging, and follow-up.

However, imaging technologies have improved greatly over the years. This is through the use of improved detector elements or the adoption of model-based image reconstruction methods for increased lesion-to-background contrast in the images of the tracer distribution. In the early years of imaging innovation, much effort went into the design and optimization of techniques that increased the sensitivity of an imaging system, while only recently, attention has shifted to optimizing imaging technologies to a higher specificity as well, so as to provide means to non-invasively assess the phenotype of tumours that present in most cases as heterogenous masses with intra- and inter-lesion variations in molecular and signaling pathways [10]. Thus, the role of imaging has remained ever so important but the demands on the imaging technologies have become more complex. The objective of this perspective is to highlight key developments of imaging methods used frequently in cancer patient management (Fig. 1) and to carve out potential areas of improvement that are foreseen in the near future.

## Positron Emission Tomography (PET) imaging and instrumentation trends

**Fig. 3:**
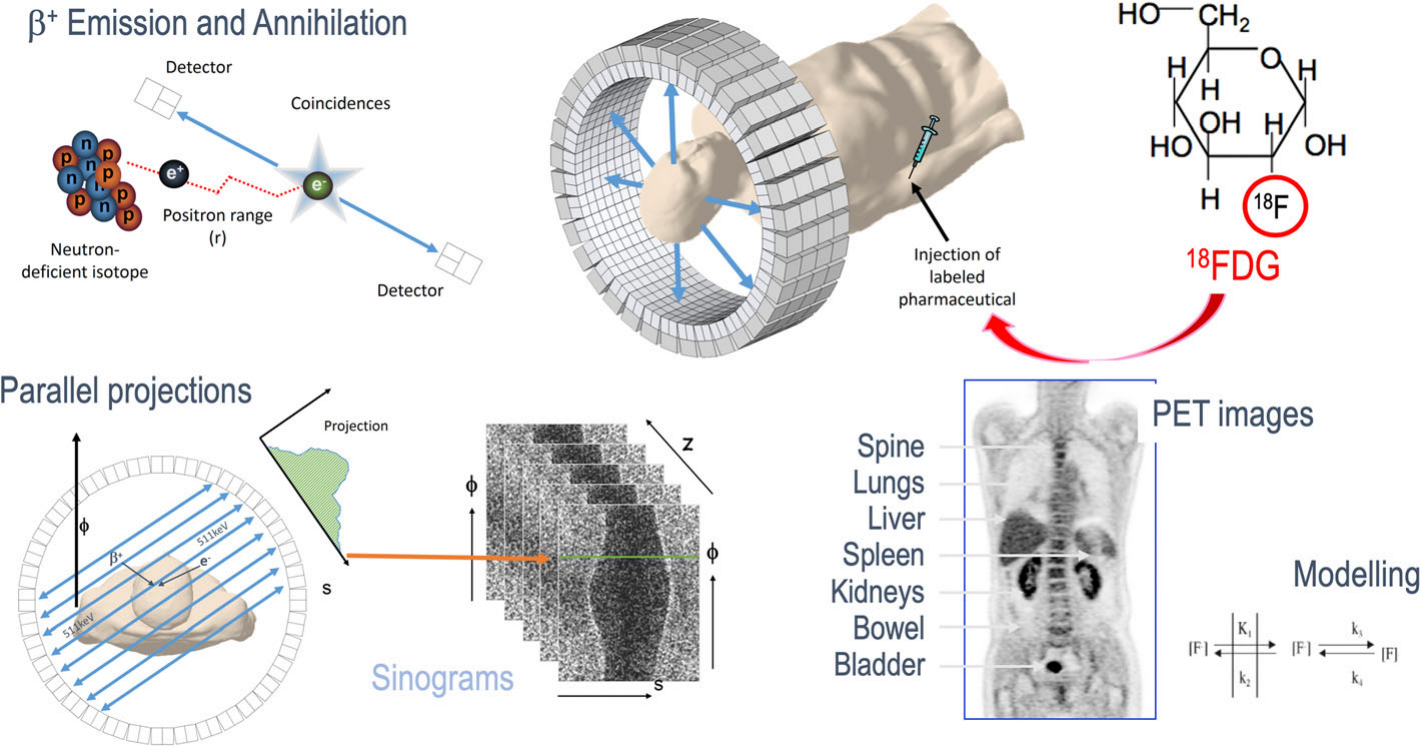
Positron Emission Tomography (PET) imaging: a neutron-deficient, radioisotope (positron emitter) is used to label a biomolecule of choice (here: glucose; Tracer,) which is injected into the patient. The resulting annihilation radiation is detected in a PET system, the data are sorted into sinograms and stored in sonograms prior to image reconstruction. In case, post-acquisition scatter and attenuation correction is performed., tracer uptake in the PET images can be quantified. Materials courtesy of David W Townsend, Singapore

PET is a non-invasive imaging technique that provides visual and quantitative information on molecular pathways. Imaging is performed post injection of a radiotracer, which – in the case of PET – is a biomolecule labelled with a neutron-deficient radioisotope [11]. During the subsequent positron decay (β+) a proton is converted to a neutron thereby releasing a positron. The β + travels a short distance - termed the positron range - before it annihilates with an electron from the surrounding tissue so as to form two annihilation photons of 511 keV each that travel in nearly opposite directions. The spatio-temporal distribution as well as the absolute concentration of the tracer can be determined through the detection and reconstruction of annihilation events (Fig. 3). Today, PET imaging has been partnered with CT and MR imaging, namely as PET/CT and PET/MR hybrid imaging systems to provide more relevant information in routine clinical practice [12].

### Status quo

With more than 6000 PET/CT systems and approximately 250 PET/MRI systems operational worldwide, the key application of PET imaging is for oncological indications [13]. Here, [18F]FDG, an [18F]-labelled glucose analogue, is the tracer-of-choice to assess the increased glucose turnover in cancerous tissues compared to normal tissue (Warburg effect). The key challenges when diagnosing cancer by means of PET are related to cancer biology and the actual ability to visually circumscribe the cancerous lesion using reconstructed PET images. Cancer biology is addressed by the a priori choice of a suitable tracer molecule to probe the existence of a suspected disease and to further phenotype it. The availability of molecular probes and the choice of the most appropriate tracer will be the focus of a companion contribution to this series.

Image resolution, which drives lesion detection, is determined by the size of the imaging detector elements, which is on the order of 3 mm in clinical PET systems [14]. The resulting imaging resolution is somewhat lower due to detrimental effects from positron range effects, which vary depending on the energy of the positron emitted, detector penetration and depth-of-interaction (DOI). It can also be influenced by image reconstruction and respiratory physiological movement during acquisition. Despite the final image resolution of PET being lower than that of MR or CT, lesions with a diameter less than the theoretical image resolution can still be detected if their contrast is sufficiently high; this is frequently seen in applications of [124I]-iodide and [68Ga]-somatostatin and -PSMA ligands due to the combination of high binding affinity and low background uptake [15]. In addition to the detrimental effects of limited spatial resolution (image blurring, partial volume effects), attenuation and scatter of the annihilation photons cause visual distortion, bias and lower contrast as well as limited count-rates, all of which contribute to increased image noise and reduced image contrast (Suppl Figure 2) [16–23].

The standard PET detector is composed of the scintillation material that stops/detects the annihilation photons and an electronic device – a photomultiplier tube (PMT) – coupled to the end of the detector material, that, in turn, transforms the detected scintillation photon into an electrical signal, which typically involves signal amplification and processing. The ideal PET scintillator material has a high stopping power – sufficient to attenuate the 511 keV annihilation photons, high scintillation light output – to facilitate accurate detection of the annihilation photons, fast decay time - to minimize deadtime, high energy resolution - to better discriminate against scattered photons, and low cost (Suppl Table 1) [14].

Recently, digital PMT – better known as silicon PMT (SiPM) – have been introduced as a replacement to analogue PMTs. SiPMs function as an ON/OFF switch, such that if scintillation light hits the SiPM an electrical pulse with sufficient amplitude is generated. With SiPM the limitations of light absorption and conversion as well as spatial resolution are drastically reduced when compared to an analogue PMT. Furthermore, their small and compact form factor and their magnetic-susceptibility tolerance make them ideal devices for use in PET/MR systems compared to their PMT counterparts, which are not MR-compliant.

The introduction of LSO- and LYSO-based detectors with their characteristic fast scintillation light decay time (Suppl Table 1) have enabled a form of PET image acquisition and reconstruction known as time-of-flight (ToF) imaging [24]. With ToF imaging, information about the minute difference in arrival time of the annihilation photons at opposing detectors is captured. This knowledge is subsequently used during image reconstruction to more precisely inform on where the annihilation event occurred within the PET field-of-view (FOV) as compared to conventional PET imaging. The knowledge about the arrival time has been shown to improve the resultant signal-to-noise ratio (SNR) of reconstructed PET images [14]. For example, a 300 ps timing resolution improves the SNR of ToF images by about 30% when compared to non-ToF images. Furthermore, larger patients benefit more from ToF imaging than smaller subjects [25].

Following the detection of the annihilation photons, corrections for self-absorption and scatter must be applied prior to image reconstruction. In PET/CT, the use of CT has been utilized for the purpose of CT-based attenuation correction (CT-AC) and scatter correction [26]. Studies have demonstrated a diagnostic benefit when performing the CT portion of the PET/CT at a radiologically-equivalent quality [27]. Some users advocate – with promising results - an adapted, mixed-phase contrast administration that can be used for both diagnostic [28] and CT-AC purpose [29].

**Fig. 4:**
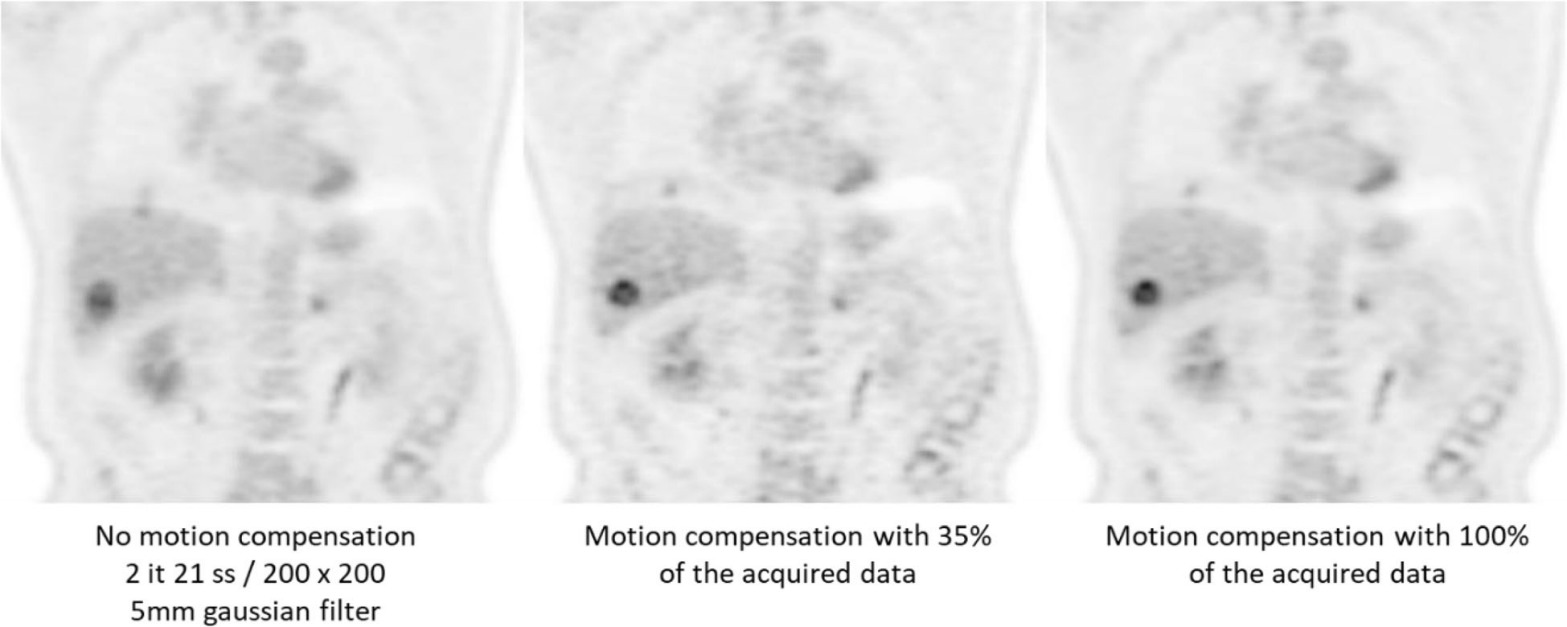
[18F]-FDG PET images of a 60-y/o male (362 MBq, BMI = 28) with colorectal cancer. Images show lewsions in the liver and right lower lung that are affected by motion-induced blurring. Left: without motion compensation, middle: with amplitude motion compensation that utilizes 35% of the acquired data resulting in noisy images but reduced motion artifact, and right: with amplitude motion compensation that utilizes all the acquired data resulting in lower image noise and motion artifact

A key challenge to high-quality and quantitative PET is physiological motion, including respiratory and cardiac motion, that may cause noticeable mismatches between the PET and CT images and blurring due of the PET images. Conspicuity of tracer uptake in lesions that are located in regions affected by physiological motion can be reduced through the use of gating or more complex motion compensation techniques. Gating describes the partitioning of a standard list-mode stream of emission data acquired during a PET scan by means of external or data-driven trigger schemes [30]. Several techniques have been developed to address these distortions, such as 4D PET/CT [31] and quiescent phase/amplitude gating [32] with various levels of success and limited adoption into routine clinical work given the complexity of the workflows (Fig. 4). Motion compensation benefits from the hardware combination of PET and MRI, since a variety of MR sequences can be exploited to estimate motion vector fields without the requirement for ancillary markers or increased radiation burden [33, 34].

**Fig. 5:**
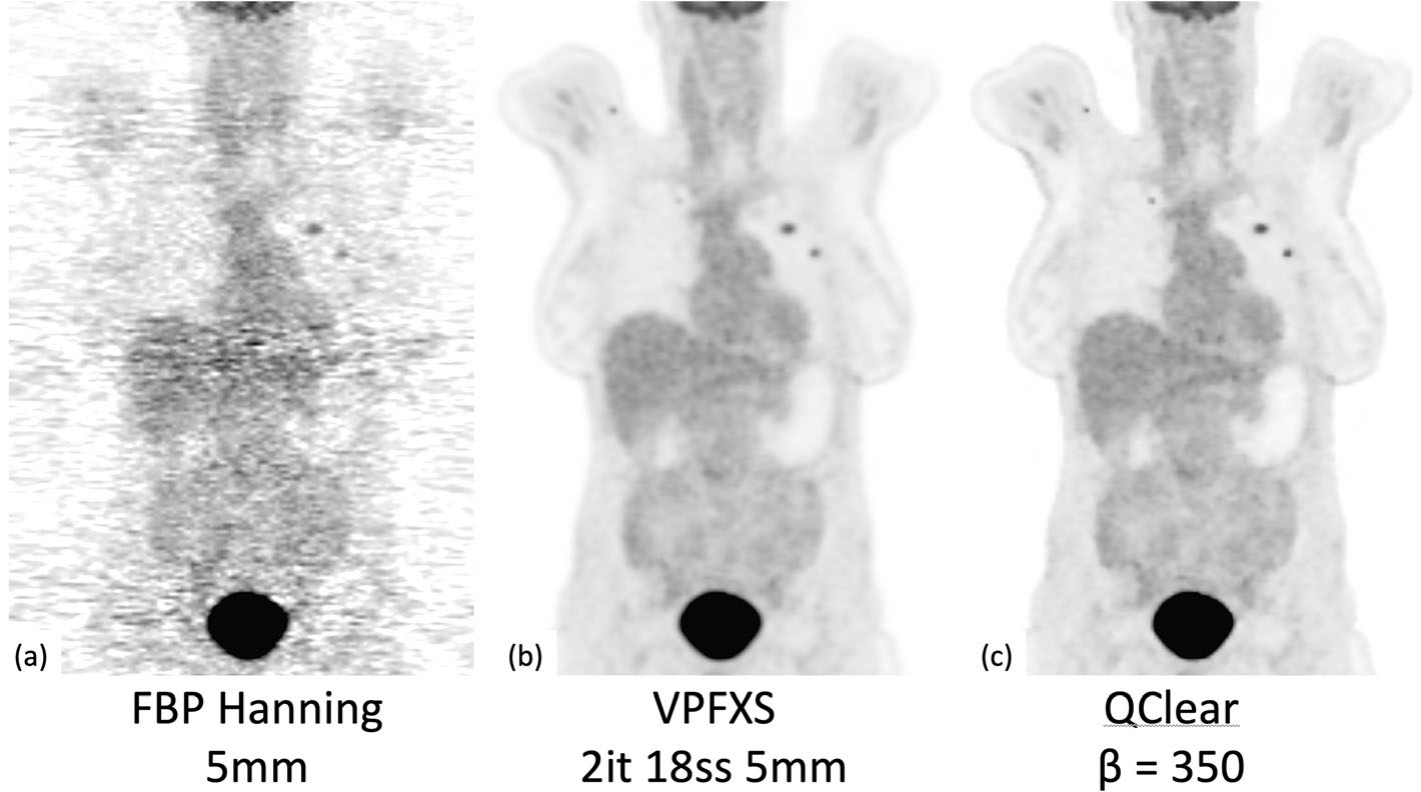
[18F]-FDG PET images of a 71-y/o female (324 MBq, BMI 22) with endometrial adenocarcinoma post hysterectomy, bilateral oophorectomy and lymph node dissection. Images show the differences in quality when using FBP reconstruction (left), iterative reconstruction (middle), and regularized reconstruction (right)

Image reconstruction in PET has evolved from the original 2D analytic techniques, such as filtered back projection (FBP) to the more recent 3D iterative reconstruction (IR) techniques, such as ordered subset expectation maximization (OSEM) (Fig. 5). Iterative reconstruction techniques have the added advantage of more accurately modelling the physics of the imaging process compared to analytical methods but they are also computationally more expensive and require a relatively longer time to reach convergence (i.e. solution). Additionally, when they reach convergence, the resultant images are characterized by high noise. In an effort to overcome this challenge, recent advances in this area have introduced regularized reconstruction approaches, which have the ability to minimize the noise while reaching convergence.

**Fig. 6:**
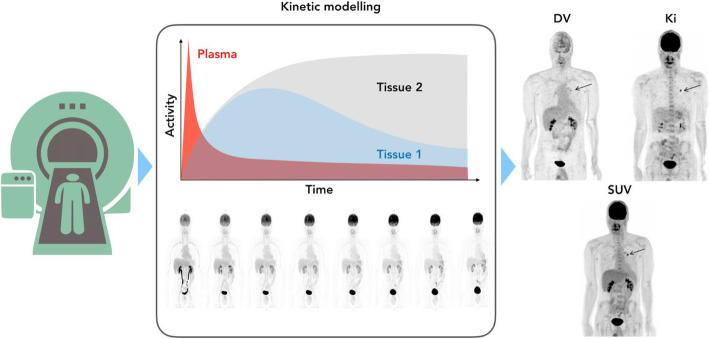
State-of-the-art PET/CT acquisitions allow for the repeated sweeps of extended axial imaging ranges during the emission scan following the injection of the tracer. These data can be reconstructed in contiguous frames (see Frame 1-8 as an example) that are used to extract time-activity curves of tissues and organs. This information, in turn, can be used to derive parametric images (e.g., k_i_ and distribution volume, DV) beyond static images that yield SUV quantification only. It is assumed that parametric image information supports more accurate diagnosis in the future. (Figure made with contributions from Rahmim (2019) and Keio Gijyuku University Hospital, Japan)

Lesions with comparatively low contrast to the surrounding tissue can be hard to detect, particularly in noisy imaging conditions originating, for example, from very short scan times, very low amounts of tracer activity injected or from scanning obese patients, which increases the scatter fraction. Thanks to the use of advanced scanner designs that allow for larger coverage of axial FOVs, new PET/CT systems permit shorter acquisition times, lower administered radiotracer amount, and easily, repeated imaging of any region of the body. These repeated whole-body PET scans can then be used to derive radiotracer uptake rate constants (K_i_-values) and, thus, estimate parametric images in addition to plain standardized uptake value (SUV) images (Fig. 6).

**In summary,** PET imaging today comes in flavours of combined PET/CT and PET/MRI with both design concepts being geared towards whole-body oncology imaging and less so full quantification. This visualization-driven approach is complemented with user software that generally limits data handling to 3D slicing of reconstructed PET images and basic quantification of static PET data.

### Status go

#### PET detectors

Most state-of-the art PET/CT systems use LSO or LYSO detector materials coupled to SiPM, and unless other, faster scintillation type detectors can be marketed, these will remain the material of choice for the years to come. Technological progress is expected in the advances of SiPM technologies, so as to making these amplifiers smaller and more robust. Furthermore, their small size should aid in the positional accuracy of the detected annihilation photons thereby improving overall image resolution and further reducing partial volume effects.

#### Time-of-flight PET

Current timing resolution of PET systems is on the order of 250-500 ps. If the timing resolution improves by a factor of 10 (25-50 ps) then there will be no immediate need for image reconstruction since the image can be directly generated as the data are acquired. Recently, an initiative was launched by a CERN-linked consortium to push for a 10 ps timing resolution in PET in order to bring together enthusiastic stakeholders to advance on this particular technological challenge for a wider adoption of PET imaging [35]. To meet this challenge, detector materials that combine the upsides of LSO or LYSO but with even faster timing resolution, have to be found and manufactured in scale. Another benefit of a faster detection system is better count-rate performance with lower dead-time effects over extended ranges of radioactivity levels, as in situations where the administered radiopharmaceutical accumulates in high concentrations.

#### Attenuation and scatter correction

It appears unlikely that CT transmission scans will be avoided in PET/CT in the future, unless techniques such as maximum likelihood attenuation and activity (MLAA) [36, 37], which can approximate both the attenuation map and the resultant image for the acquired data, or Machine and Deep learning-based AC methods for PET imaging applications are developed. However, little data is available as to the robustness of these approaches and their performance in the presence of varying tracer distributions, morphological alterations or high-density implants.

#### Motion compensation

Data-driven gating (DDG) techniques have been introduced to address patient motion induced image blur [38]. DDG benefits from using fast PET detectors that yield a high SNR to allow the extraction of the motion waveform from the background noise. It is anticipated that with the elimination of external devices to record the motion cycle, the motion compensation workflows will become much easier to use and will be adopted routinely in the PET clinic. In return, PET image quantification will be more accurate and patient management will be more precise [39].

#### Image reconstruction

Assuming the 10 ps challenge will not be answered in the next few years, iterative reconstruction (IR) will prevail (Fig. 5b). IR methods will be improved with more advanced system models that describe various aspects of the hardware and the subjects (motion, tracer distribution etc) more accurately (Fig. 5c). For example, CT attenuation maps might be compromised by metal artefacts, truncation, and contrast media; or in the case of PET/MR attenuation maps may not be available altogether since MR images do not represent tissue attenuation. In this regard, new reconstruction algorithms, such as MLAA and artificial intelligence along with machine learning approaches that use deep learning algorithms hold a big promise in addressing these challenges [40–42]. The performance of these techniques is currently being evaluated under various clinical conditions such as low count-density (shorter scan times, low activity) and varying radiopharmaceutical biodistributions.

#### New system design concepts

Traditionally, PET systems have been designed as cylindrical structures with detectors dotting the circumference of these structures and oriented towards the central axis of the cylinder. More recently, systems with longer axial lengths have been introduced. By placing detector modules abutting next to one another along the axial extent of the scanner, lengths of 20 cm, 25 cm and 26 cm have been achieved on commercial systems from GEHC and Siemens Healthineers respectively, with one manufacturer (United Imaging) achieving 194 cm [43]. This increase in axial extent has two key advantages. First, it increases sensitivity, which can be translated into higher image quality or traded for a reduced injected activity, and, second, it facilitates increased patient throughput by reducing acquisition time. A total-body PET/CT scan can then be performed in one minute, or less and with a significantly reduced amount of administered radiotracer, which consequently reduces patient radiation exposure, thereby potentially transforming the use of PET imaging from a diagnostic tool to a screening tool. Other advantages of total-body PET with longer axial extents include the ability to perform dynamic imaging of multiple organs simultaneously, which enables the study of the bio-distribution and pharmacokinetics of new radiopharmaceuticals and disease states with images representing true biological parameters, such as tracer uptake rate constants (K_i_), blood flow, or receptor density rather than just semi-quantitative values, such as standardized uptake values (SUV) (Fig. 6).

PET/MR systems will continue to evolve albeit slowly when compared to PET/CT with improvements in attenuation correction, truncation, and image uniformity in organ-specific or whole-body applications. Their adoption into routine clinical care will primarily depend on the identification of clinical applications that leverage the advantages of MR (compared to CT) while complementing the diagnostic information gained from PET [44, 45].

**In summary,** PET imaging systems will come primarily in combinations with CT for whole-body oncology applications, unless, key drivers for the adoption of fully-integrated PET/MRI are found, in which case relatively more PET/MRI systems could be installed. PET detector elements can now be manufactured with very fast scintillation materials and produced in modules fit for use in PET/CT and PET/MRI combinations. These modules, when designed with SiPM, should help improve spatial resolution and reduce partial volume effects. The use of ToF and advanced image reconstruction algorithms will create flexibility in imaging protocol designs and help push the image quality even in low-count situations, which helps in signifying even early time-point measurements shortly after tracer injection, and with parametric imaging. Motion compensation will likely become key for the advancement of the methodology, which, if resolved, will open PET for a wider range of applications and help push it into PET-guided therapy planning. In short, the authors hypothesize a primary push for sensitivity and full quantification, which is further supported by fast and higher-resolution measurements (Table 1).

**Table 1:**
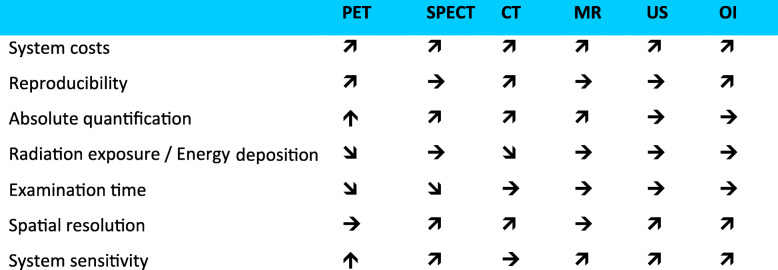
Progress indicators of methodological and commercial efforts towards improving key imaging technologies and widening their applications in clinical cancer care. *System costs* relate to costs of manufacturing a next-generation imaging system. *Reproducibility* relate to efforts made towards increasing reported accuracy of imaging results from the same system generation and across different platforms. *Absolute quantification* stands for means to extract absolute numeric values from image-based measurements. *Radiation exposure* describes exposure of subjects to ionizing radiation (or other means of energy deposit, such as in case of MRI). *Examination time* describes duration that subjects need to remain motionless in an imaging platform for the acquisition to take place. *Spatial resolution* is the measured resolution of the images. *System sensitivity* relates to both volumetric sensitivity of an imaging system and diagnostic sensitivity through the use of alternative tracer and contrast applications. The direction of the progress indicators, as relative metrics, corresponds to the consensus of the authors regarding the progress being made in any of these efforts within the next 5 years, or so

## SPECT imaging and instrumentation trends

As with PET, SPECT is a non-invasive molecular imaging method that generates tomographic images of functional and metabolic pathways in the body. Unlike in PET, SPECT is based on the use of radiotracers that are labelled with radioisotopes that decay via the emission of single or multiple photons of discrete energies. Given the lack of annihilation effects, SPECT relies on the use of physical collimators to generate projection data for localization of the SPECT tracer. SPECT has only recently been made quantitative through the adoption of correction and image reconstruction approaches [46].

### Status quo

The availability of SPECT and its ability to image a range of metabolic signals has made it commonplace in oncology imaging for both diagnosis and disease monitoring. From the identification of bone metastases [47] to the assessment of malignant thyroid conditions [48], SPECT is a common feature of oncology imaging. More recently, with the growth of theranostics [49], radionuclide therapies, and image-based dosimetry associated with therapies, there has been renewed interest in SPECT imaging technology.

SPECT systems are typically based on a pair of large (540 mm × 400 mm) energy selective scintillation detector arrays, with positional information derived from the use of lead or tungsten constructed collimators and the Anger principle [4]. The technology is well established and has changed little since its inception in 1956. Nevertheless, driven by the need for more compact and innovative designs initially for cardiac SPECT [50], but more recently for breast [51] and general oncology imaging [52], semiconductor detectors have been introduced as an alternative to traditional scintillation detector systems. The most common semiconductor material used for SPECT is Cadmium Zinc Telluride (CZT) [53]. In addition to its compactness and with its direct translation of gamma photon to signal, CZT detectors offer superior energy resolution compared to traditional gamma cameras [54]. This translates into improved scatter rejection, in addition to sensitivity and contrast gains [55]. CZT is also incorporated into SPECT systems as individual pixelated detectors, which allows the system to work at higher count rates than traditional gamma camera designs [53].

**Fig. 7:**
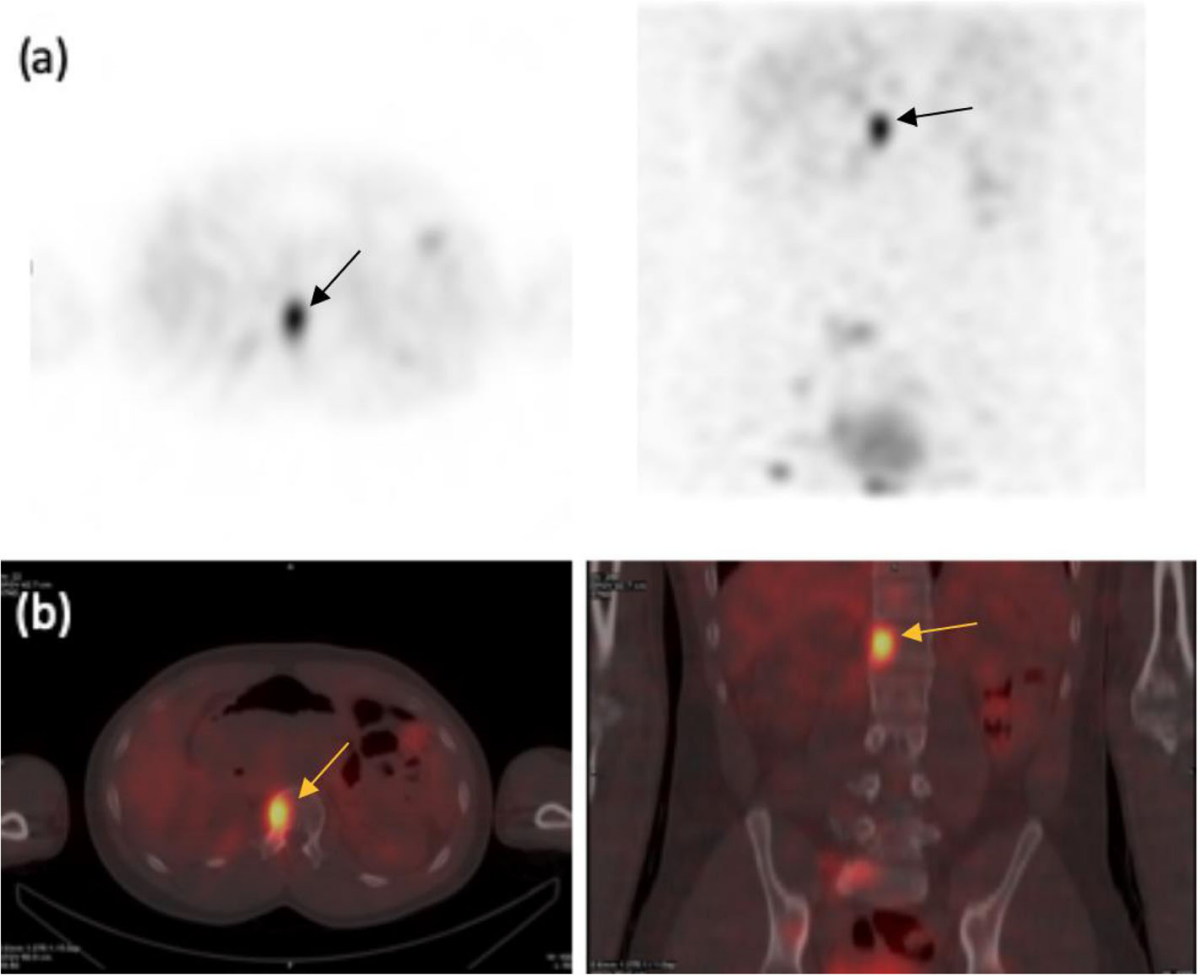
Iodine-123 MIBG image of a NET metastasis shown with (**a**) SPECT and (**b**) SPECT/CT. SPECT only images of the metastasis do not provide anatomical information, while the SPECT/CT image provides both localization and complementary CT information

The introduction and development of dual-modality SPECT/CT systems has been another step change in SPECT [56]. Transmission imaging in SPECT was originally introduced to correct for photon attenuation in cardiac studies, but was soon superseded by much quicker SPECT/CT systems. In addition to attenuation correction from SPECT/CT systems, the advantages of having localization and other complementary CT information superimposed with SPECT was quickly realized [56–58]. Co-localisation has been particularly beneficial for oncological applications of SPECT, where for example, CT can help determine whether bone lesions are cancerous or degenerative in nature, and also with radiopharmaceuticals such as mIBG, where the anatomical information available in the SPECT image can be limited (Fig. 7).

In parallel to the growth of CT-based attenuation correction [59], iterative reconstruction algorithms have replaced traditional FBP reconstruction [60]. One advantage of iterative reconstruction is the ability to integrate corrections into the image reconstruction, which provides superior solutions than correction pre or post reconstruction. This approach of integrating corrections into the reconstruction process started with attenuation and scatter correction, but has been developed further by the introduction of resolution modelling into the reconstructions system model [61]. The noise handling nature of resolution modelling is revolutionizing SPECT acquisition protocols because it can provide significant (~ 60%) reduction in acquisition time for the same level of image quality provided by traditional reconstruction methods [62]. This is a great benefit for some oncology-based SPECT studies where acquisition times can be 30 min, or longer. Alternatively, resolution modelling can be used to improve image quality for the same acquisition time as is used traditionally.

**In summary,** SPECT with its wide range of available radiopharmaceuticals, its low cost and ready availability provides oncologists with images of metabolic disease for diagnosis and disease monitoring. Recently, the technology has also seen growth driven by theranostic applications. The introduction of dual-modality SPECT/CT has helped overcome some the challenges of SPECT-only image interpretation, and developments in detector technology and image reconstruction have helped improve image quality. Nonetheless, imaging times are still long and fully-quantitative SPECT imaging is challenging – unlike in PET where it is commonplace.

### Status go

#### Hardware

One of the major drawbacks in current SPECT technology is its limited sensitivity, which results in much longer examination times compared to PET. As such, scan times of 40 min to 70 min are typical if multiple SPECT acquisitions are required to image the area of interest. Therefore, for SPECT to prosper, more rapid scan technology is required. A promising method that has been adopted in cardiac SPECT, but which has not yet translated to oncology SPECT yet is the use of very high-sensitivity collimators [54]. One of the key limitations of such collimators is the compromise of spatial resolution. However, if septal penetration is adequately controlled, there is the opportunity to use resolution modelling techniques to recover much of the lost resolution.

A second method of increasing the sensitivity of SPECT systems is to improve the detector geometry by considering alternative designs to the traditional two rotating planar detector arrangement. The return of 3, 4, or more rotating detectors can bring significant gains, but this solution is problematic because of the degraded spatial resolution seen with traditional parallel hole collimators at the distances necessary to accommodate the detectors. However, the growing availability of resolution modelling within the image reconstruction will allow the re-adoption of this design concept for SPECT. Complex collimator designs, such as multiple pin-hole [52] and slit-slat collimators [63], could also bring sensitivity gains while maintaining acceptable spatial resolution. Such efforts could be matched with improvements to intrinsic spatial resolution and semi-conductor readout [64]. While complex collimator designs are an area of interest, whether this technology can be extended to larger fields-of-view rather than small field-of-view application, such as in the brain, is to be determined.

#### Detectors

Semiconductor detector technology based on, for example CZT, is also moving into oncological SPECT. Initial models have been based on traditional dual detector design with the improved energy resolution offering some clinical advantages [65]. More recently ring detector systems have been produced, which again improve the geometric and overall sensitivity of SPECT systems, while maintaining spatial resolution by using dynamic detector motions [66]. There is no doubt that this ring detector technology will bring benefit to organ-specific imaging, such as the heart, where dynamic tomographic imaging has already shown to be beneficial, and the brain where dynamic imaging and motion correction could offer significant advantages. The ability to easily perform whole-body tomography for oncology applications, such as bone SPECT, and mIBG SPECT will benefit staging and disease progression applications in much the same was as seen in PET. Large volume SPECT will also bring benefits with dosimetry applications, by ensuring that dosimetry of tumours and organ at risk can be performed in a timely manner. However, a disadvantage of current semiconductor detectors is the usable energy range, which is typically below 200 keV. While acceptable for imaging with Technetium-99 m and Iodine-123 radiopharmaceuticals, this threshold is not compatible with common theranostic radionuclides. For example, the threshold sits below the preferred 208 keV imaging window of Lutetium-177 [67], and well below the upper 364 keV energy window of Iodine-131. To allow faster SPECT acquisitions using a ring detector, semiconductors optimal for these higher energies may be required, or possibly using scintillation crystals with traditional or solid-state readout [64, 68].

#### Hybrid SPECT

Another area where we may see hardware changes in the future is in alternative hybrid SPECT imaging. While SPECT/CT already exists, there are also developments in SPECT/MR [69]. In addition to MR providing much better soft tissue contrast for SPECT localization than is available with CT, as with PET/MR there are also opportunities to use MR information to manage patient motion [70], improve image reconstruction [71], and to delineate areas of interest, which in turn could be used to perform partial volume correction of the emisison data [72].

#### Data processing

**Fig. 8:**
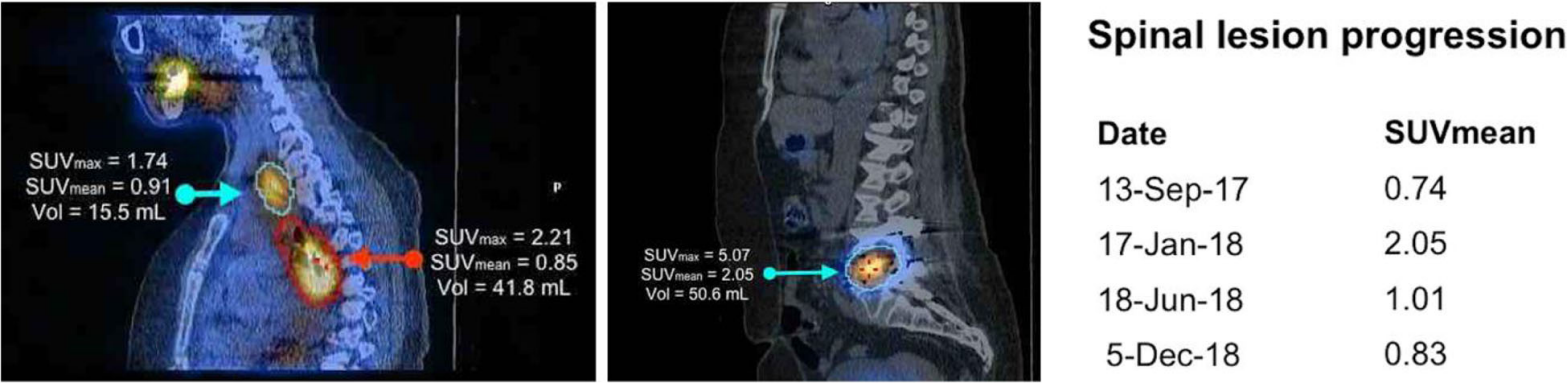
Contemporary SPECT software allows the direct derivation of voxel values and regions in terms of activity concentration or SUV as shown in this post Iodine-131 ablation SPECT/CT scan. Using this quantitative information (e.g., in the spinal lesion), it is possible to track the metabolic responses to therapeutic interventions over time

The development of contemporary correction and image reconstruction algorithms has brought with it the ability to use SPECT as a quantitative imaging modality in much the same way as PET [73]. Measurement of in-vivo activity concentrations or standardized uptake values (SUV) are now possible and may become the norm in future SPECT imaging (Fig. 8). Indeed, as with PET, by incorporating segmentation algorithms, measurements will be developed further to help derive metabolic tumour volumes and total tumour burden to better characterize the overall disease status [74]. The basis of this technology already exists [75], and applications of the technology are being investigated for both cross-sectional diagnosis prognosis and staging, as well as longitudinal treatment response and disease progression indications [46].

#### Reconstruction and corrections

Although CT-AC may become the norm with very few SPECT-only studies performed, there will be a drive to reduce CT radiation exposure by the use of improved iterative CT reconstruction and artificial intelligence algorithms [76–78]. Indeed, similar techniques will also be applied in SPECT, where available count statistics in oncology imaging are often limited, and there is an inability to extend already long scan times. Improved iterative reconstruction models using penalized likelihood [79, 80] and improved resolution modelling will help, particularly if they overcome overshoot ‘Gibbs’ artefacts [81]. Additionally, developments with scatter correction algorithms will help move the technology from subtractive techniques, which are inexact and increase image noise [82] compared to those incorporated into image reconstruction [60], which do not increase image noise. Artificial intelligence algorithms are already being developed that can improve the levels of noise seen with low count statistic studies, and it is envisaged that in the next 5-10 years such algorithms will make issues from low photon flux imaging less problematic [83].

An issue that can come from the necessary long acquisition times of SPECT is that of patient motion. In SPECT, spatial resolution is typically between 8 mm and 12 mm, so small levels of patient motion are not problematic. However, larger levels of motion will degrade image quality, and frequently require the patient to be rescanned. To overcome these issues, motion detection and improved motion correction software is being developed, which will help both identify motion and correct data where possible, while also alerting the technologist when the data is unusable [84]. It is also envisaged that that there will be significant development in future years in the routine application of Monte Carlo based image reconstruction techniques [75]. Currently algorithms are very slow and mostly used in research labs, but there is huge potential in this form of reconstruction.

#### Theranostics

While SPECT already has an important role in theranostics, there has been a recent surge of new theranostic applications using Lutetium-177 labelled peptides in neuroendocrine tumours, and PSMA in prostate cancer [85–89] with many other therapeutic areas in development by commercial partners [90]. Key to making the best use of this technology is the need to understand the radiation dose given to various tumour targets and organs at risk. Quantification of radiopharmaceutical uptake together with accurate segmentation of tumours and organs of interest are central to the derivation of accumulated activity curves required for radiation dosimetry. Currently, performing this work is challenging, and often requires additional imaging, characterisation, and home-grown image processing algorithms [91]. Supported by tools to assist in the calibration and standardisation of quantitative SPECT systems, the practice of dosimetry in molecular radiotherapy will become widespread, supporting the vision of personalised precision medicine with theranostics.

#### Radiopharmaceuticals

A more detailed synopsis of radiopharmaceuticals will be given in an accompanying paper by Blower and colleagues. Nevertheless, with the penetration of SPECT in the market and the wide availability of SPECT radionuclides, there is the possibility of moving PET radiopharmaceuticals to SPECT - as seen with Technetium-99 m labelled TOC, PSMA and glucose analogues [86, 92, 93]. Clearly, this is an opportunity for low- and middle-income nations where PET and cyclotron technology may not be readily available, while for high-income countries it may depend on whether the sensitivity and spatial resolution limitations of current SPECT technology can be overcome.

**In summary,** SPECT hardware designs will evolve rapidly, which together with developments with collimation and image reconstruction will improve the sensitivity of SPECT systems and reduce examination time (Table 1). There is also the potential for improvements in sensitivity and spatial resolution, which will result in SPECT imaging with better quality than we have today. While technological changes will have an impact, it is with the modality becoming truly quantitative that the use of SPECT that will grow. The current growth trajectory of theranostics and the ability to quantify SPECT data in terms of SUV will lead to a greater demand in SPECT for both radiation dose assessment, and the ability to monitor treatment response. Beyond theranostics, quantitative SUV SPECT will help in the interpretation of SPECT data and will lead to more precise diagnosis using the technology.

## Computed Tomography (CT) imaging and instrumentation trends

Computed tomography (CT) is an anatomical imaging method. Here, a narrow beam of X-rays is directed to the subject and quickly rotated around the body, thereby producing transmission signals. These signals are processed and reconstructed to form cross-sectional images of the subject under investigation. CT is a tomographic imaging method that generates contiguous axial images, which can be digitally stacked to form 3-dimensional, high-resolution images of the investigated area. Oncologic diseases can be depicted through alterations of standard anatomy or alterations of dynamic processes, such as restricted intravenous contrast enhancement.

### Status quo

#### X-ray transmission

Diagnostic CT imaging is based on the measurement of X-ray absorption from a large number of different view angles across the patient. State-of-the-art CT systems offer a sub-second rotation time and are capable of acquiring multiple slices simultaneously, thereby covering a longitudinal range of 2 cm to 16 cm during a single rotation. The most dominant scan mode in CT is the spiral scan mode, which is a continuous data acquisition combining a continuous gantry rotation with a simultaneous translation of the patient through the gantry [94]. The trajectory of the X-ray source relative to the patient is a spiral, or helical trajectory. Spiral acquisitions yield the lowest possible scan times at the highest possible image quality, whereby the latter is due to the high symmetry of the spiral trajectory. In general, scan times range from far below 1 s to several seconds. Nearly all scans can be conducted within a single breathhold, thereby avoiding respiratory motion artefacts.

#### Quantification

CT systems offer an isotropic spatial resolution of about 0.5 mm and, therefore, are best suited for anatomical imaging. The physical property that is displayed in CT is the distribution of the linear attenuation coefficients. For convenience, the values are linearly transformed to become integer-valued Hounsfield units (HU) in a way such that air corresponds to − 1000 HU, water to 0 HU, and soft tissue and fat to about 50 HU, and -70 HU, respectively. However, tissue differentiation by means of HU alone remains challenging, particularly for a range of soft tissues; while soft tissues exhibit similar X-ray attenuation properties, their difference is noticeable more clearly on MR images. Therefore, most CT scans in oncological imaging require the administration of an iodinated contrast agent to achieve sufficient image contrast between the vessels, perfused organs and tumours relative to the surrounding soft tissue and fat. The contrast injection is typically synchronized with the acquisition of the CT scan to ensure sufficient contrast in the organ of interest during the short time of scanning.

#### Functional CT

Functional properties can be assessed with CT by performing dynamic perfusion scans. During perfusion protocols, a 4D acquisition is performed by repeatedly imaging the same body region in time lapses of 3 s to 5 s for about 30 s post administration of the contrast agent. Using the so-called “time attenuation curve”, important hemodynamic parameters can be derived, such as the blood flow, the blood volume, or the permeability surface area product. Such functional parameters have been shown to correlate with histopathology and therapy response [95–97].

#### Ionizing radiation

Despite the lack of solid arguments for a linear no threshold (LNT) model, fears associated with radiation-induced risks have led to a wide number of efforts to reduce the dose in CT. These include the introduction of tube current modulation, automatic exposure control, automatic tube voltage selection, more powerful X-ray tubes that allow for thicker pre-filtration, highly-integrated detectors with less internal noise, multi-dimensional adaptive raw data filtering, as well as iterative image reconstruction algorithms [98]. These techniques make more efficient use of the X-ray dose and help reduce the X-ray dose while preserving image quality. Alternatively, such methods can also be used to improve the image quality without increasing the patient dose. The latter may be of importance for oncological imaging where frequently subtle contrast changes in small lesions need to be detected, and where patients may undergo a large number of follow-up scans. The need for dose-conscious CT imaging is typically amplified when scanning young patients.

#### Detectors

Suppl. Table 2 lists important properties of state-of-the-art CT systems. For example, maximum tube power at low tube voltages helps lower patient exposure or boost image quality of contrast-enhanced scans, since the iodine in contrast agents exhibits a k-edge at low energies. Most CT vendors provide the possibility to reconstruct the images at grids larger than 512 by 512 pixels, so as to use the full spatial resolution capabilities for large field-of-view reconstructions, which helps improve sensitivity in lung imaging or imaging bone metastases.

#### Dual-energy CT (DECT)

**Fig. 9:**
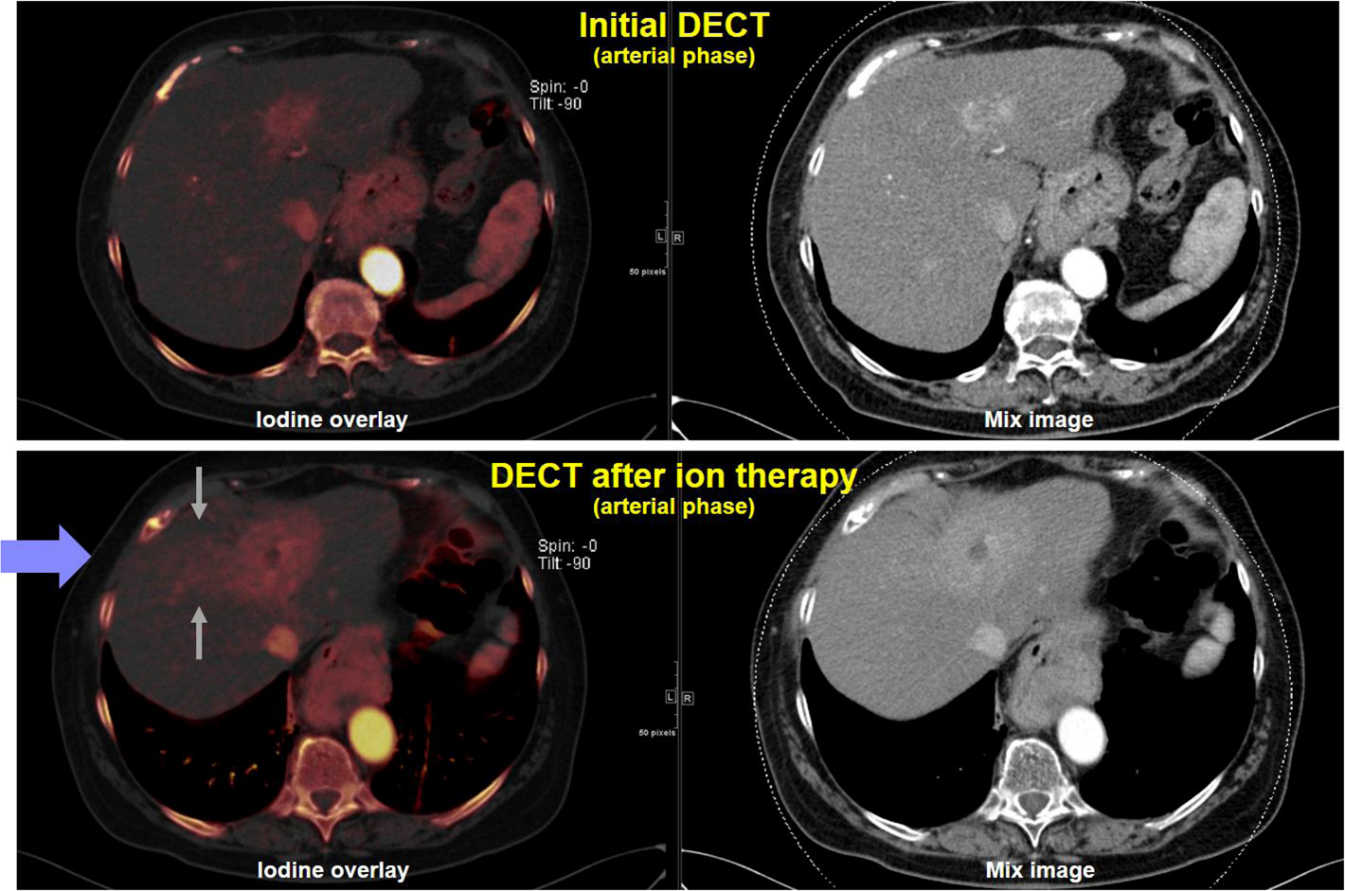
Patient with HCC before and after hadron therapy. DECT helps delineate iodine uptake within the tumour and the recurrent lesions. The contrast accumulations (gray arrows) in the irradiation field (blue arrow) can only be deduced from the iodine map, thus, improving differentiation of HCC and surrounding healthy liver. Data courtesy of the Division of Radiology of the German Cancer Research Center (DKFZ)

DECT exploits differences in the energy dependency of the attenuation coefficients and thus allows to selectively display materials that might appear at the same CT value in single-energy CT. DECT is can be realized by including a dual-source CT, a CT with fast tube voltage switching, a CT equipped with a sandwich or dual-layer detector, or a CT with different prefiltration on different parts of the detector array [99]. Approaches that combine two separate CT scans are also available, but suffer from motion in-between the scans. Applications of DECT include the selective display of iodine and bone, the characterization of kidney stones, the reduction of artefacts and the quantification of materials (Fig. 9). Advantages of DECT for oncological imaging have been reported for tumour detection and lesion characterization [100] and for surrogates of perfusion measurements [101, 102]. Well-designed scan protocols help distribute the X-ray dose across the two X-ray spectra, such that the total dose remains similar to that of a single energy CT [103, 104].

#### Metal artifact correction (MAR)

Metal artefacts are the most dominating type of artefacts in CT. In many cases, metal artefacts prevent accurate diagnosis of lesions that are close to or in-between metal objects. Moreover, the artefacts degrade the attenuation correction needed for PET imaging [105]. Metal artefacts are mainly caused by the strong beam-hardening of dense objects, related to the strong attenuation of metal. Due to the corruption of the projection data behind the metal the only way to reduce such artefacts is to replace the raw data in the vicinity of the metal implant [106]. Standard metal artifact reduction algorithms (MAR) are based on inpainting techniques, such as FSNMAR [107], IMAR [108], or OMAR [109].

**In summary,** CT systems comprise up to 100 detector rows and allow for routine acquisitions with spatial resolution on the order of 0.5 mm within scans that take less than a second to a few seconds at dose value ranges of sub mSv to 15 mSv. CT images are highly quantitative and reproducible and come with very good contrast resolution. The most dominant artefacts are addressed by dedicated correction algorithms. Dual-energy CT acquisitions are available in the mid-range and premium systems.

### Status go

#### Photon counting

**Fig. 10:**
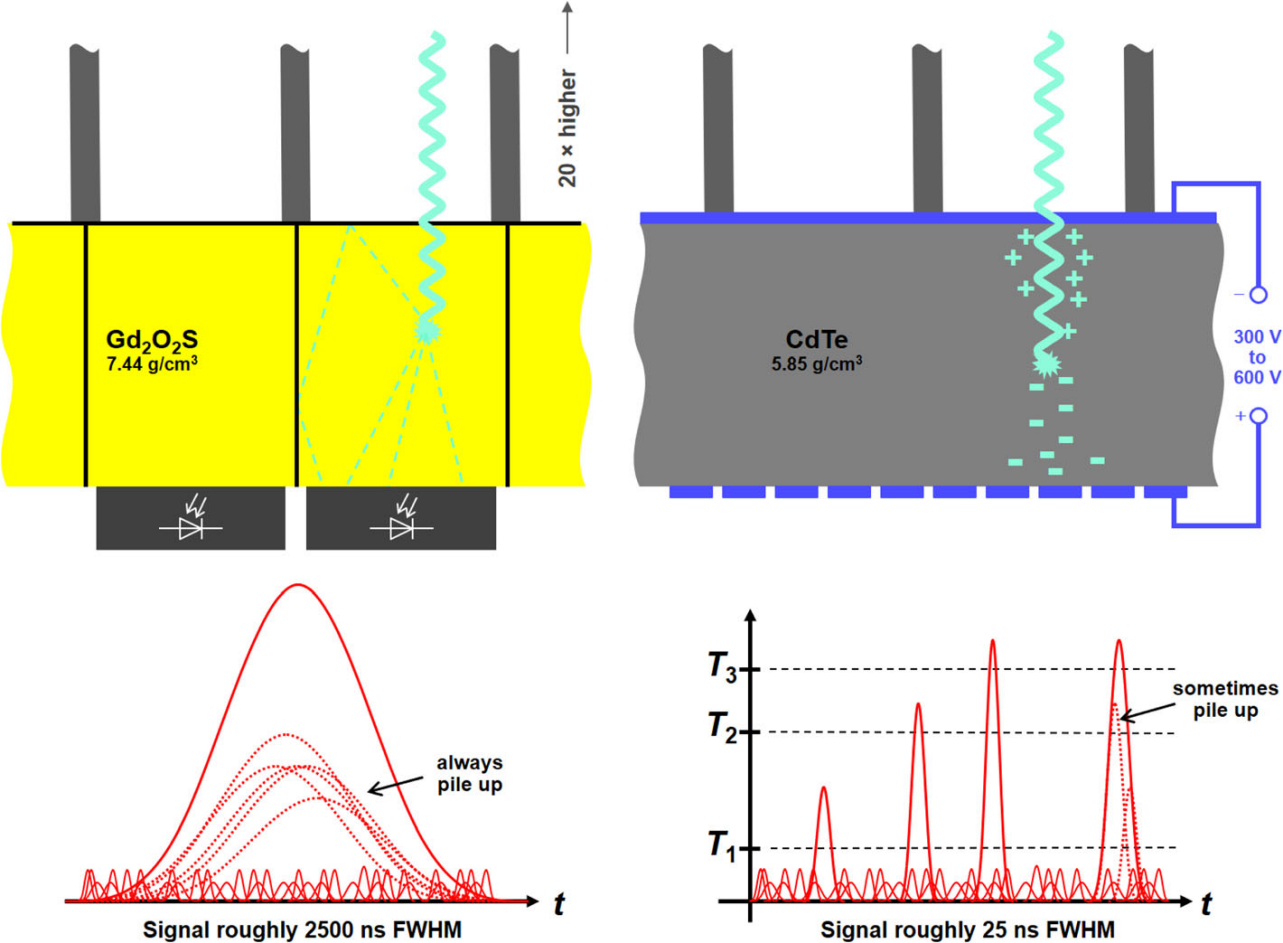
Conventional energy integrating (EI) detectors convert the X-rays into an electric signal indirectly (left). Future photon counting (PC) detectors convert each X-ray photon into an electric peak directly (right). The peak of the PC detector is short enough in time to permit counting single photons. The peak height gives information about their energy. To minimize pile up the PC detector pixels are much smaller than the EI pixels

Future CT systems may be equipped with novel X-ray detectors (Fig. 10). Currently-available detectors are indirect converters that convert X-ray photons into visible light, which is then converted into an electrical current. The next generation of CT systems may utilize photon counting detectors, which directly convert the X-ray photon into an electric signal. In contrast to the indirect converters, the bell-shaped electric signal that is generated in the photon counting detector pixel following the registration of an X-ray photon, is very short in time – short enough to be able to count a photon before the next photon arrives. Moreover, the height of the peak can be measured. Since it is proportional to the photon energy, photon counting detectors will be energy-selective, thus, inherently producing spectral information without the need to generate two different X-ray spectra (DECT) [110]. It is assumed that a future photon counting CT may distinguish four energy bins, thus, the yielding information similar to that from a conventional CT scan performed at four different tube voltages.

Apart from the inherent capability of performing spectral separation, photon counting detectors also promise to yield images with lower noise levels or to acquire images at lower patient exposure levels. Furthermore, iodine contrast will be increased with photon counting detectors. Conventional detectors give a high weight to high energy photons and a low weight to low energy photons, including those that are close to the k-edge of iodine (33 keV). In contrast, photon counting detectors weight low and high energy photons equally and, thus, yield improved iodine contrast.

#### Machine learning (ML)

**Fig. 11:**
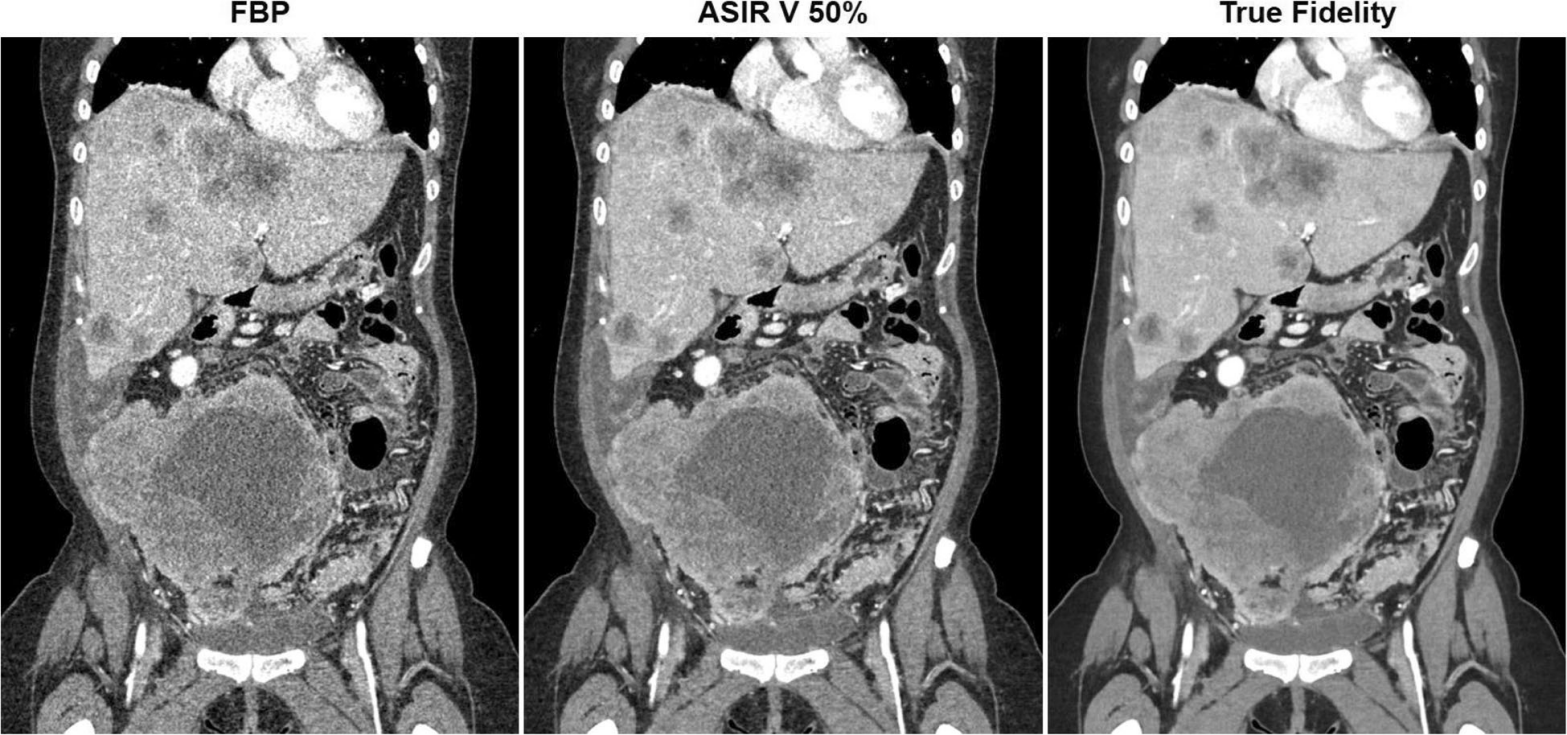
Patient exam reconstructed with FBP (left), an iterative algorithm (middle) and a deep learning (DL)-based approach (right). The potential of DL-based CT image reconstruction is illustrated by the significantly lower noise levels while with preserving image detail and increasing visibility of low contrast structures. Courtesy of GE Healthcare

ML has entered the field of CT imaging and will gain importance, not only for computer-aided diagnosis and big data evaluation, but also for CT image formation. For example, image reconstruction may benefit from deep learning approaches to help reduce patient exposure or improve image quality [78, 111] (Fig. 11).

**In summary,** CT technology is evolving towards images with spectral information at significantly higher spatial resolution and greatly reduced noise. These development will positively impact oncology imaging applications, such as assessing the thorax, or the characterization of bone metastases. Further, the characterization of low contrast lesions will be improved. The added spectral information, which will be available on demand, may benefit from the development of new contrast agents. These advances will likely find their way into PET/CT systems to improve attenuation correction and increase soft tissue contrast even for low-dose CT scans (Table 1).

## Magnetic Resonance Imaging (MRI) and instrumentation trends

MRI is founded on the principle of nuclear magnetic resonance (NMR), whereby spin-carrying nuclei under a strong static magnetic field (B_0_) can be excited with a weak oscillating radiofrequency (RF) field (B_1_) and absorb its energy [112–114]. The observation that the relaxation time constants of the NMR signals are different between normal and cancerous tissues preceded the advent of MRI [115]. In MRI, magnetic field gradients (G) are used to make the frequency and phase of the spin precession spatially dependent and, thus, produce an image of the signal emitting spins [116, 117]. Compared to other imaging modalities, such as CT and PET, MRI has the intrinsic advantage of providing multifaceted and excellent image contrast, especially for soft tissues, and for not utilizing ionizing radiation. MRI can also be performed in any orientation without relying on postprocessing image reformatting. Conversely, MRI has an intrinsic limitation in signal-to-noise ratio (SNR), and because most MRI schemes rely on acquiring one line of k-space at a time, it has been relatively slow.

### Status quo

#### Magnets and gradients

In the magnet technology, the superconducting magnet, active shielding, and “zero helium boil-off” are a few significant milestones that have helped shift MRI from being low field (0.1 – 0.2 T) in the early days to the present day of high-field systems (1.5 – 3.0 T) as the standard. A strong motivation for the development is the increased intrinsic SNR, which is approximately proportional to B_0_. Unfortunately, higher magnetic field strength also entails technical challenges, including higher magnetic susceptibility and higher RF field dielectric effects. On the other hand, the development of faster and more powerful gradients, including better eddy current management with actively shielded gradients and real-time compensation strategies, has largely been motivated by fast imaging, such as echo planar imaging, fully-balanced steady-state imaging, and fast spin echo imaging. The successful implementation of these fast imaging techniques has brought to fruition of novel clinical applications, such as blood oxygen level dependent or BOLD functional MRI, and diffusion weighted imaging (DWI), which has found application in early detection of stroke and providing better characterization of tumours.

#### RF coils

Similar to magnet and gradient innovations, the RF technology for MRI has also been undergoing a steady stream of development. In the early days of MRI, a single “one-size-fits-all” RF coil was often used for both transmit and receive. The phased-array technology [118] is a major step forward in MRI as it provides simultaneously the large coverage of a volume coil and the superior SNR of a small surface coil. The phased-array coils have also enabled parallel imaging [119–121] and simultaneous multi-slice (SMS) imaging [122], which greatly speed up the MRI acquisition by combining the different spatial sensitivity profiles of the different coil elements with magnetic field gradient for spatial encoding. Similarly, phased-array coils also enable parallel RF transmit [123], which has the potential for tailoring the excitation profiles (e.g., for spatially focused excitation or reduced dielectric artefacts). For these and other advantages, many present-day MRI scanners carry a wide variety of phased array coils and are equipped with up to 128 or more simultaneous RF channels.

#### Oncology protocols

**Fig. 12:**
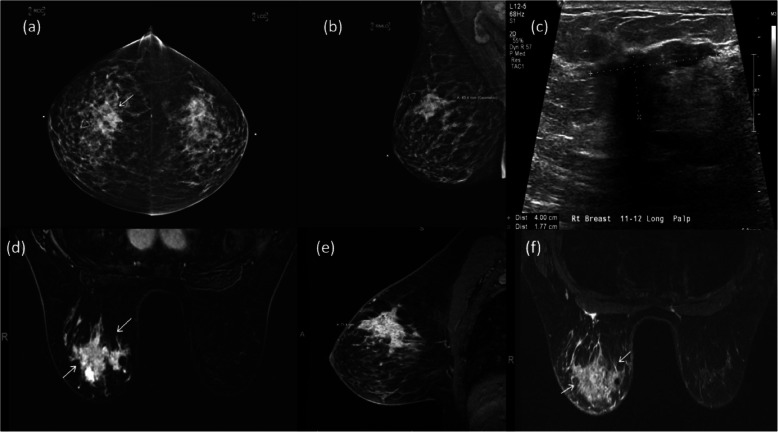
A 59-y/o female presented with palpable mass of right upper breast. Biopsy revealed intraductal carcinoma (IDC) grade III. (**a**) Digital mammography cranial-caudal (CC) views of both breasts. Notice the lesion (arrows) was poorly delineated due to dense breast. (**b**) Mediolateral oblique (MLO) view indicates longest dimension of lesion to be 4.3 cm. (**c**) Under targeted US, the lesion was seen as ill-defined hypoechoic mass with longest dimension of 4 cm. Axial and sagittal post-contrast, fat-suppressed dynamic contrast enhanced (DCE) MRI shows the lesion of a much larger extension at 7.3 cm and avidly enhancing (**d** and **e**, arrows). (**f**) Axial fat-suppressed T2-weighted image shows the large carcinoma (arrows) as hyperintense lesion involving most of the upper part of the breast. Images courtesy of Maia Rauch, MD, PhD

From its very beginning, clinical applications, such as in cancer, have always been the underlying driving force for the development of MRI. The versatility of MRI is reflected in many different types of images that can be generated with the different scan protocols, pulse sequences and image reconstruction algorithms. Among them, T1-weighted and T2-weighted images, in conjunction with Gadolinium-based contrast agents, have been the mainstay for most oncological MRI applications, especially for the detection, characterization, and staging of the tumours (Fig. 12). The development of the fast spin echo (alternatively known as turbo spin echo, or RARE) pulse sequence [124], which employs a train of rapidly refocused echoes after each excitation to fill multiple lines of k-space, greatly reduces the acquisition time for T1-weighted or T2-weighted images. Many magnetization preparation schemes have also been developed to further enhance the utility and versatility of a basic pulse sequence, such as inversion recovery for fluid attenuation (FLAIR) [125] or chemical shift selective saturation (CHESS) for fat suppression [126].

#### Multi-parametric MRI

In addition to stationary tissues, MRI can also be used for imaging moving blood or cerebral spinal fluid (CSF) in MR angiography (MRA) to detect and depict vascular abnormalities. For this application, several MRA methods have been developed and are in routine clinical use. Contrast-enhanced (CE) MRA relies on the injection of a Gd-based contrast agent to shorten the T1 of the blood, thus, creating enhanced signal for the blood relative to the background tissues. Time-of-flight (TOF) MRA, on the other hand, relies on the inflow enhancement of the blood signal and various saturation techniques to suppress the signal from the stationary background tissues. Although less robust and usually of lower spatial resolution, TOF-MRA does not require contrast agent injection.

Compared to other imaging modalities, a unique feature of MRI is that its signals have complex rather than scalar values. The velocity of the moving spins, such as from blood, can be encoded into the phase of the MR signals through the application of magnetic field gradients. Phase-contrast (PC) MRA is based on this principle and compared to CE- or TOF-MRA, has the advantage of being able to quantitatively measure the blood flow [127]. PC-MRI is also the basis of MR Elastography [128], which can measure the tissue stiffness and has been successfully applied for staging liver fibrosis and differentiation between benign and malignant tumours. Another important application where the phase of the MRI signals is successfully manipulated is Dixon imaging [129], by which water and fat in the tissue can be separated with a combination of modified data acquisition and postprocessing. Dixon imaging, in combination with the segmentation of bone and air by a zero-TE (ZTE) pulse sequence, has also been useful for creating the attenuation maps that are needed for accurate PET reconstruction in the combined PET/MR systems [130].

**Fig. 13:**
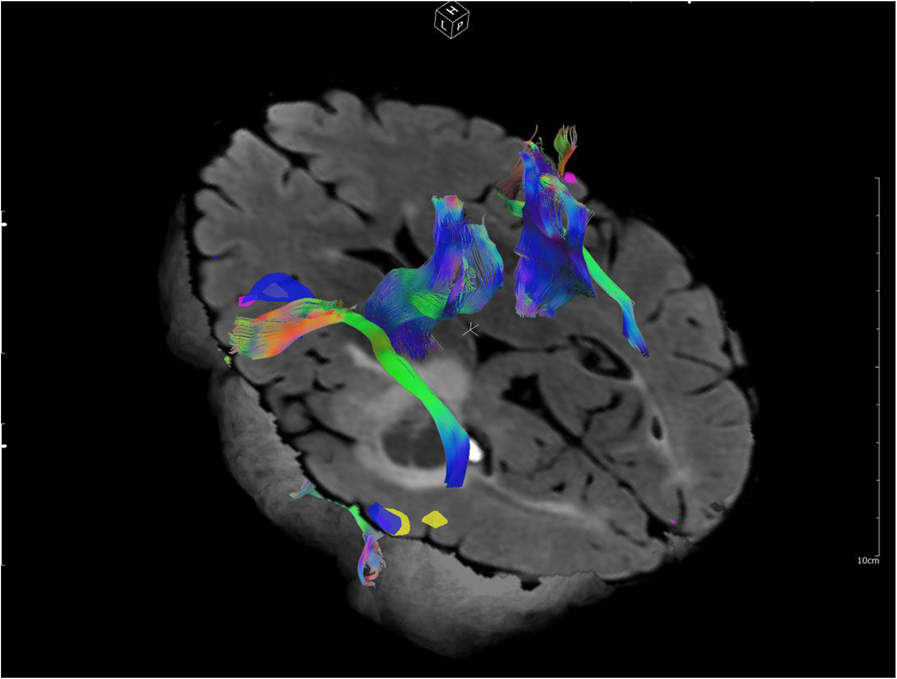
Functional MRI (fMRI) and diffusion tensor imaging (DTI) tractography for presurgical evaluation of brain tumour resection. Significant fMRI activations were detected in both anterior (Broca’s) and posterior (Wernicke’s) areas of the language network in the left hemisphere when the patient was performing language tasks (blue: word generation, pink: category naming, yellow: sentence completion). Critical fibre bundles including corticospinal tract (motor) and arcuate fasciculus (language) were generated with fibre tracking of the DTI data. (Images are courtesy of Ho-Ling Liu, PhD)

In addition to the conventional T1, T2, and proton density-weighting, several alternative contrast mechanisms in MRI have been developed and found their value for imaging of cancer. Diffusion-weighted imaging (DWI) encodes the Brownian motion of water molecules into the signal loss through the application of large diffusion-sensitizing gradients. Unlike the image contrast by T1 and T2, which is generally related to “water content”, diffusion contrast is sensitive to changes in the intra- and extra-cellular structures and density. Further, DWI images can be used to generate an apparent diffusion coefficients (ADC) map, which can serve as a quantitative marker for tissue characterization without requiring an exogenous agent. In cancer applications, restricted diffusion and low ADC have in general been found to correlate with cell malignancy and aggressiveness [131]. DWI also serves as the basis for diffusion tensor imaging (DTI) [132], which measures the diffusion anisotropy of the underlying tissues and has been useful for preoperative planning by depicting the relationship between brain tumours and adjacent white matter fibre tracks (Fig. 13).

Chemical exchange saturation transfer (CEST) imaging [133] is an MRI contrast mechanism that relies on the magnetization transfer [134] and chemical exchange between the protons of water and the protons of some labile macromolecules, such as the mobile proteins or peptides. The detection of these macromolecules with CEST is indirect, yet with a sensitivity that is enhanced by orders of magnitude compared to the detection of water. Interestingly, CEST can measure not only the concentration of these macromolecules but also the pH-level of their environment because the latter is dependent on the rate of magnetization transfer and chemical exchange. Since the accumulation of the macromolecules and pH-level of their environment that are measurable by CEST are sensitive to the cancer metabolism and progression, there has been an intense interest in investigating the potential of CEST for a variety of cancer applications, including for improved detection, characterization, and treatment response assessment.

**In summary,** MRI has established itself as a modality of choice in the detection, characterization, and staging of cancers of many types, including those of the brain, spine, liver, prostate, rectum, and breast. Many technical limitations of MRI of the early days (e.g., speed, SNR, and motion artefacts) have become increasingly well-managed. Thanks to the excellent soft tissue contrast and the growing number of useful contrast mechanisms, MRI is an indispensable tool for imaging of cancer.

### Status go

#### Hardware

Wide-bore scanners with light-weight and more anatomy conformal phased array RF coils will become increasingly available, making MRI more patient friendly and less time-consuming to operate. Hardware enhancements with anatomy-dependent adaptive shimming, such as in the areas of C-spine/neck and shoulders, and more accurate and easy-to-use motion monitoring/management devices are also expected to substantially improve the image quality and reduce the scan-to-scan variability.

#### Dual-modality combinations with MRI

**Fig. 14:**
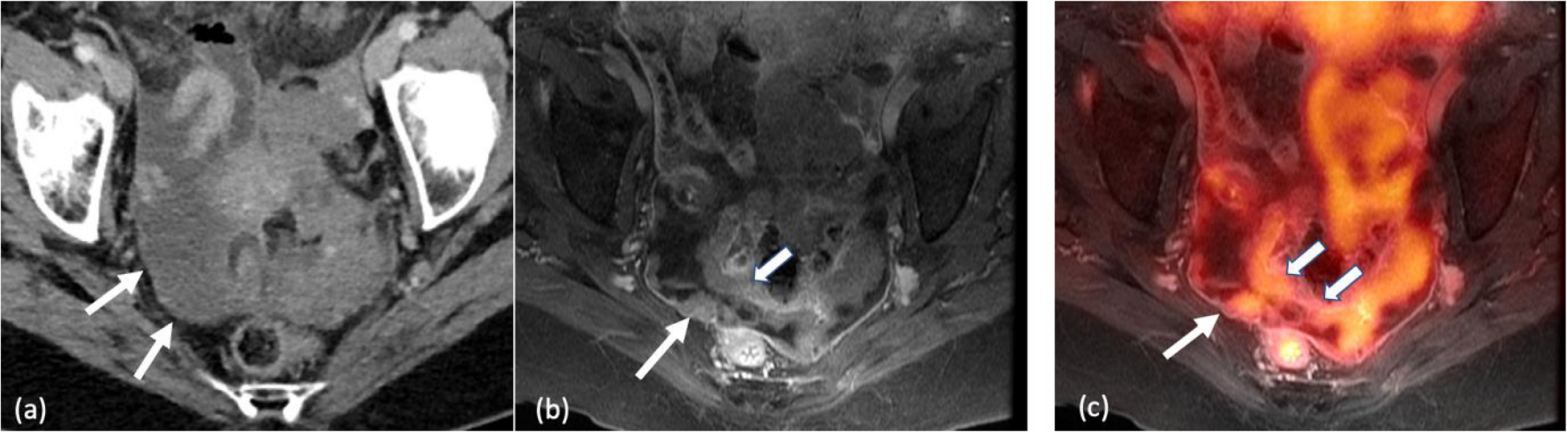
A 65-y/o female with ovarian cancer. (**a**) Axial contrast-enhanced CT shows ascites and peritoneal enhancement (arrows) of concern for peritoneal disease. (**b**) Post-contrast T1-weighted MRI shows enhancement and nodularity in the pelvis along the peritoneal lining (arrow) and thickening of the sigmoid colonic wall (fat arrow) of concern for peritoneal disease. Serosal involvement of the sigmoid colon may require colonic resection. (**c**) Axial fused PET/MR shows [18F]FDG-avid lesions along the pelvic peritoneal lining (arrow) and along the sigmoid colonic wall (fat arrows) representing peritoneal disease and serosal involvement of the colonic wall, which may require resection of the sigmoid colon. Images are courtesy of Priya Bhosale, MD

MRI has been integrated successfully as a major component in a couple of dual-modality systems, such as PET/MR [135, 136] and MR-Linac [137]. PET/MR, in particular, will be used to explore the value of quasi-simultaneous acquisitions of metabolic information from PET and morpho-functional information from MRI (e.g., T1, T2, DWI, perfusion, CEST) for tumour characterization [45]. It should be recognized, however, that key applications remain to be identified and established in clinical routine. There is evidence that the high soft tissue contrast of MR and its simultaneous acquisition with PET data are useful for accurate local staging and treatment assessment of several types of tumours, such as gynaecological and prostate tumours, and that, therefore, fully-integrated PET/MRI could potentially be regarded as advantageous over PET/CT (Fig. 14). With regard to MR-Linac, the superior soft tissue contrast and ability to continuously image a moving tumour (e.g., in the liver or lung) and its surrounding anatomy by MRI can be leveraged to guide and optimize the treatment of the tumour by external beam radiation and minimize the radiation damage to the normal tissues. The potential for functional assessment of the tumours by MRI is also an advantage over the traditional and more established CT-guided radiotherapy.

#### Speed and SNR

Several novel and exciting techniques are emerging to further increase the speed and SNR in MRI. Compressed sensing [138], which employs the image sparsity, random undersampling, and non-linear iterative reconstruction, enables MRI beyond the Nyquist limit without a major resolution loss. In conjunction with parallel imaging, compressed sensing is poised to become an important acceleration option for many MRI acquisition. MR Fingerprinting [139], which allows quantitative mapping of multiple tissue parameters simultaneously with a single acquisition and postprocessing pattern recognition with the aid of a pre-established signal dictionary, has the potential to become a useful tool for tissue parameter quantitation and detection of a target disease change with high sensitivity.

#### Machine learning

**Fig. 15:**
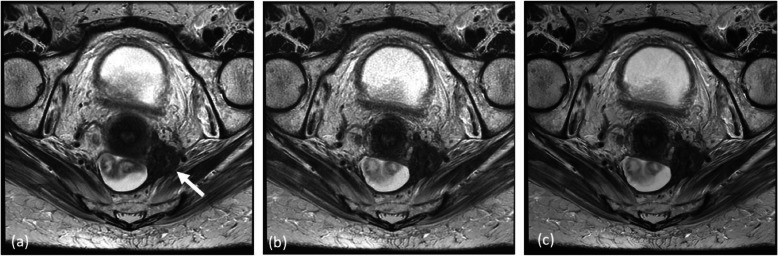
3-T high-resolution axial T2-weighted images of a patient with adenocarcinoma of rectosigmoid junction. (**a**) Image acquired with a standard clinical protocol with a number of signal excitations (NEX) of 2, with arrow showing a mass with irregular infiltrative margins. (**b**) Image acquired with an identical scan protocol and patient setup, except with the NEX reduced to 1 and thus scan time reduced by half as compared to (**a**). An expected reduction in SNR is noted. (**c**) Image generated from a DL-reconstruction algorithm using the same acquisition as in (**b**). Notice the restoration of SNR without loss of resolution compared to (**b**)

As for other imaging modalities, artificial intelligence (AI) and machine learning (ML) have the potential of bringing about the most disruptive changes to MRI [140]. Although still considered at its infancy, AI/ML has demonstrated an array of remarkable applications in MRI data acquisition and image reconstruction (e.g., for speed acceleration, resolution enhancement, and generation/synthesis of multiple image contrasts including across the different modalities), in image processing (e.g., automatic artefacts removal and denoising without blurring) (Fig. 15), and in image analysis and direct diagnosis (e.g., automated tumour and anatomy segmentation, image co-registration, and tissue parameter estimation without relying on empirical modelling, classification of tumour type, grade, and treatment response). AI/ML has also been successfully applied for practice efficiency improvement through protocol determination based on short-text classifications, for substantially reduced Gd-dosage in contrast-enhanced MRI without a noticeable reduction in image quality, and for generating synthetized images used for PET/MR attenuation correction. There is little doubt that the list of AI/ML applications in MRI will grow rapidly. However, it is also important to note that many challenges remain for these applications to be translated into clinical practice, among them cross-site validation of a trained model/algorithm, clinical workflow integration, and regulatory hurdles.

**In summary,** MRI is expected to continue to evolve and expand in technical scope and clinical applications. In addition to becoming more patient- and user-friendly, MRI is poised to break some fundamental boundaries (such as in speed, sensitivity, and artefact removal) with several emerging technologies, including compressed sensing and deep learning (Table 1). The improved image quality and the accuracy and reliability of the quantitative MRI measurements will be highly valuable in better tumour characterization and in better longitudinal assessment or cancer prognostication.

## Ultrasound (US) imaging and instrumentation trends

Ultrasound (US), also referred to as sonography, is a non-invasive imaging method that employs sound waves to generate images of the inside of the body. US is based on high-frequency sound waves that are emitted from a transducer that is placed on the body surface over the volume of interest. The same transducer is used to receive the sound waves, which are reflected by the target. Depth-encoded US images can be generated by determining the run time of the reflected sound waves within the tissue. Because of the ease-of-use and widespread availability, US is used frequently as the first diagnostic imaging modality during a patient work-up. This is true for investigations of most soft tissues, except the lungs and the brain. The key advantages of this imaging modality are the lack of radiation, its real-time imaging capability, low cost and high mobility of the devices.

### Status quo

US-imaging strongly evolved over the last decades [141]. State-of-the-art transducer technology supports the recording of the higher harmonics of reflected ultrasound pulses (harmonic imaging), thereby, improving image quality, in particular for deeper layers of tissue. Significant advances, with respect to the field-of-view and the transition from 2D to 3D data assessment, have been achieved by introducing moving transducer arrays inside handheld instruments, e.g., for breast imaging [142, 143]. Real-time 3D data acquisitions are made possible with phased array volumetric ultrasound scanners [144]. However, specific design aspects of the transducer matrix including cross-talk between elements and size and number of elements render the fabrication of matrix transducers difficult and costly [145].

**Fig. 16:**
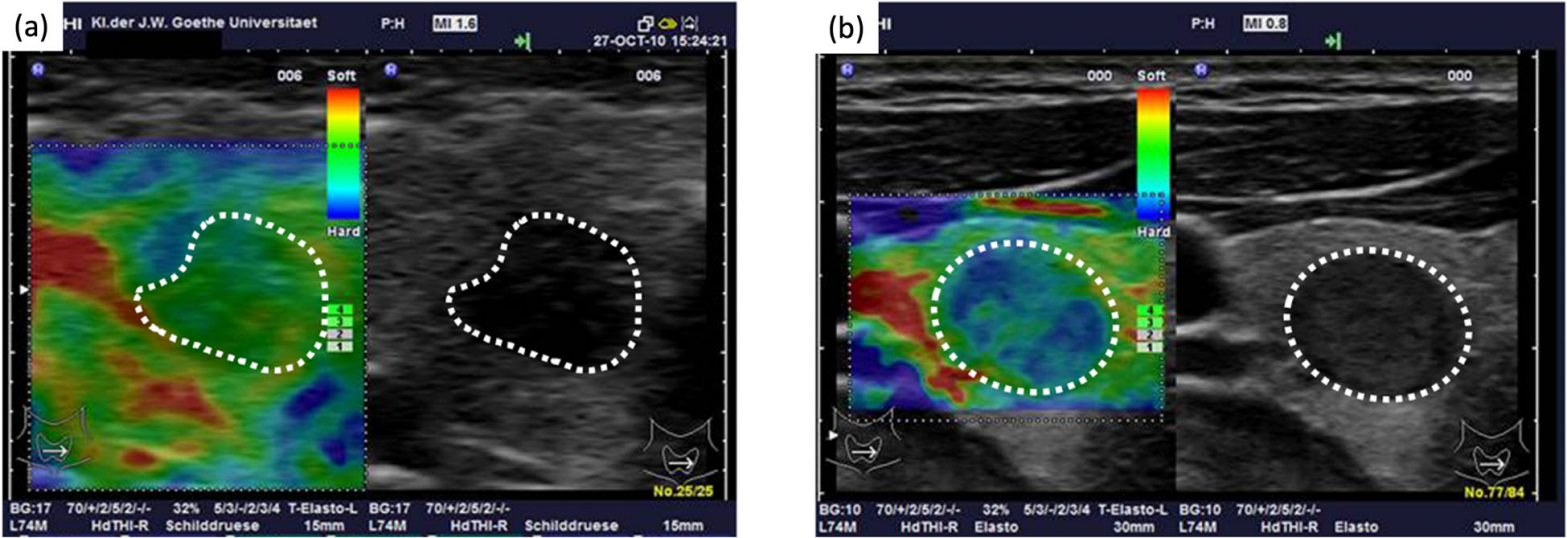
Ultrasound elastography. Elastography (colour-coded) and B-Mode images of a thyroid adenoma (**a**) and a papillary carcinoma (**b**) with the lesions encircled in white. The elastography images show clear differences and, thus, help characterize the carcinoma through reduced elasticity, as depicted by the blue regions, which correspond to a lower elasticity (image adapted from [150])

Next to volumetric imaging, also other technologies, such as ultrasound elastography (USE) have been developed to extend the field of ultrasound applications. Transducers capable of strain or shear wave imaging support the non-invasive characterization of the mechanical properties of tissues. Given the differences in tissue elasticities in specific pathologies, such as organ fibrosis and tumours, USE has became a valuable diagnostic tool for the differential diagnosis of healthy and diseased tissues. For example, USE of the breast can be applied to differentiate between benign and malignant lesions, given that malignant lesions appear more heterogeneous and are stiffer than surrounding healthy tissues [146, 147]. The combination of Brightness-mode (B-mode) US with USE further improves the specificity to detect vital malignant tissue and reduces the number of required biopsies [148, 149]. Chronic thyroiditis and malignant tumours are associated with higher stiffness, thus, making USE a valuable tool for malignancy assessment of nodules and biopsy guidance (Fig. 16**)** [150].

In addition to the evaluation of mechanical tissue properties, the aberrant vasculature in tumours is of high interest for tumour characterization and therapy monitoring. Doppler methods, which use deformations of sound waves reflected from moving elements in the blood to visualize blood flow, enable a detailed vascular characterization not only for assessing plaques and stenoses in larger vessels, but also to detect larger neoplastic vessels. However, at clinically applied frequencies the very fine vascular networks can still only be detected with contrast-enhanced US (ceUS) scans. Today, a variety of commercial and clinically approved contrast agents is available [151]. These agents are based on microbubbles with a size of 1-3 μm, which can be separated into a gas core and a shell material [151]. Microbubbles can be destroyed and, thereby, detected by highly energetic US (e.g., Power Doppler imaging) or non-destructively detected by harmonic imaging by their non-linear responses, e.g., using amplitude modulation and pulse inversion sequencing modes [152–155]. In particular, ceUS is used in clinical routine for the characterization of liver lesions [156], thereby significantly improving tumour characterization as well as supporting early and specific therapy response assessment [156, 157]. Consequently, many clinical guidelines now mention ceUS imaging as a diagnostic option [158–160].

Next to diagnostic applications, US can also be used for therapeutic applications. Therapeutic high-intensity focused ultrasound (HIFU) applications were developed that are often combined with diagnostic US imaging [161]. Focusing US pulses to a defined location in tissue can lead to a regionally defined energy deposition. Depending on the settings, thermal or mechanical effects can be induced, known as thermal ablation or histotripsy. So far, HIFU is approved by the FDA for six indications, including uterine fibroids, benign and malignant prostate lesions, essential tremor, tremor dominant Parkinson’s disease, and bone metastases [162].

**In summary**, low cost and lack of radiation make US a frequently used imaging modality for first-line investigations, intermediate follow-up investigations during pharmaceutical therapies and interventions. In this context, its anatomical and vascular imaging capabilities are of particularuse and applied together.

### Status go

#### Three-dimensional US imaging

Advances in the development of micromachined ultrasound transducers might enable to overcome current limitation in the number and positioning of elements in matrix transducers [163]. However, the control of far more than 256 channels in parallel is necessary to make use of the full potential of these transducers, and to maximize image quality. Next to a higher number of active transmit and receive elements, the image quality could also benefit from image reconstruction using 3D datasets.

#### Ultrafast US imaging and US localization microscopy

**Fig. 17:**
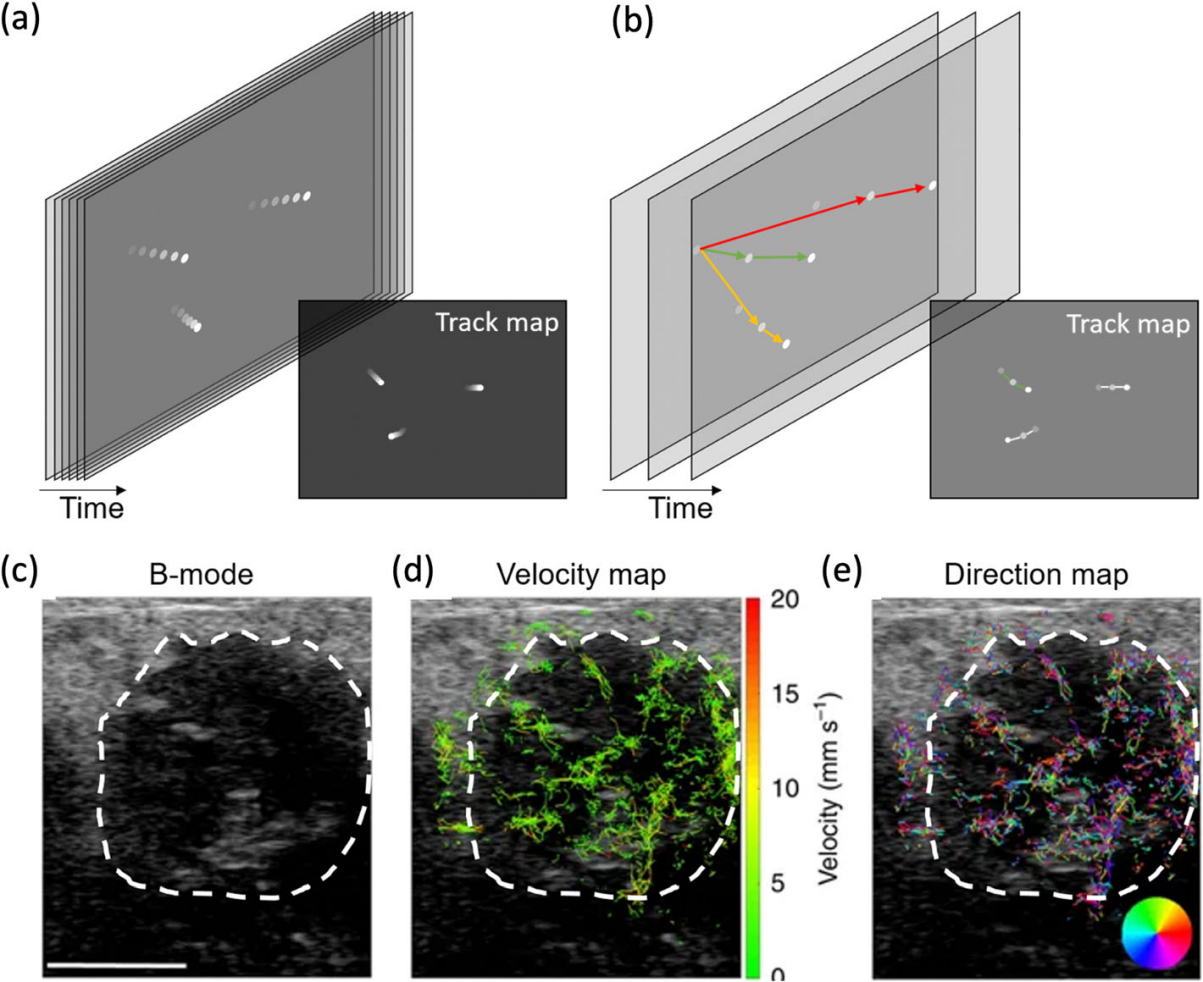
Super-resolution ultrasound. (**a**) Ultrafast Ultrasound Localization Microscopy (uULM) uses ultrasound devices with very high frame rates to achieve reliable tracks of microbubble movements. (**b**) In motion-model Ultrasound Localization Microscopy (mULM) tracks between voxels that are enhanced by microbubbles are generated, and their likelihood is calculated using a motion probability model. Three potential combinations are schematically displayed, with the green track symbolizing the highest probability. In uULM and mULM the diameter of the tracks is defined to be close to the size of microbubbles, and super-resolution images of the vasculature are generated. (**c**) B-Mode image of a breast cancer in a patient (encircled in white). (**d**) mULM based velocity map of a breast cancer in a patient. (**e**) The corresponding flow direction map, deriving from mULM analysis, provide important information about vessel maturity (image adapted from [166])

Conventionally, the elements of an ultrasound probe are excited sequentially, whereby the number of lines determines the frame rate and thus, the speed of the image generation. Novel ultrafast imaging systems are capable of computing all lines in parallel [164]. Therefore, an ultrasound image can be computed from a single transmit pulse. However, image generation is limited by the propagation speed of sound in the medium. Using ultrafast scan technologies in Doppler, the motion of individual blood cells or microbubbles can be tracked [165]. Displaying these tracks in the size of the blood cells, images of the vasculature are obtained that exceed the resolution limit of the scanner (Ultrasound Localization Microscopy, ULM). As an alternative to ultrafast data acquisition, contrast-enhanced US helps generate super-resolution images at lower frame rates if motion models are applied to assign the detected microbubbles to vascular tracks [166]. This method is termed motion-model Ultrasound Localization Microscopy (mULM). Generally, ULM enables the analysis of the vascular architecture (e.g., vessel length and branches) with an interpolated spatial resolution of a few micrometers. At the same time, functional information (i.e. blood flow velocity) can be acquired. Opacic et al. first successfully applied mULM in patients and demonstrated responses to therapy in breast cancer patients (Fig. 17**)** [166]. There remains room for improvement regarding the adaptation of the algorithms used in pre-clinical experiments to the clinical situation, where scanners work at different frame rates, ultrasound frequencies and in cases of more significant motion [167]. Once this is achieved, clinical studies need to prove the additional value of super-resolution ultrasound for various applications, such as malignancy assessment or treatment monitoring.

**Fig. 18:**
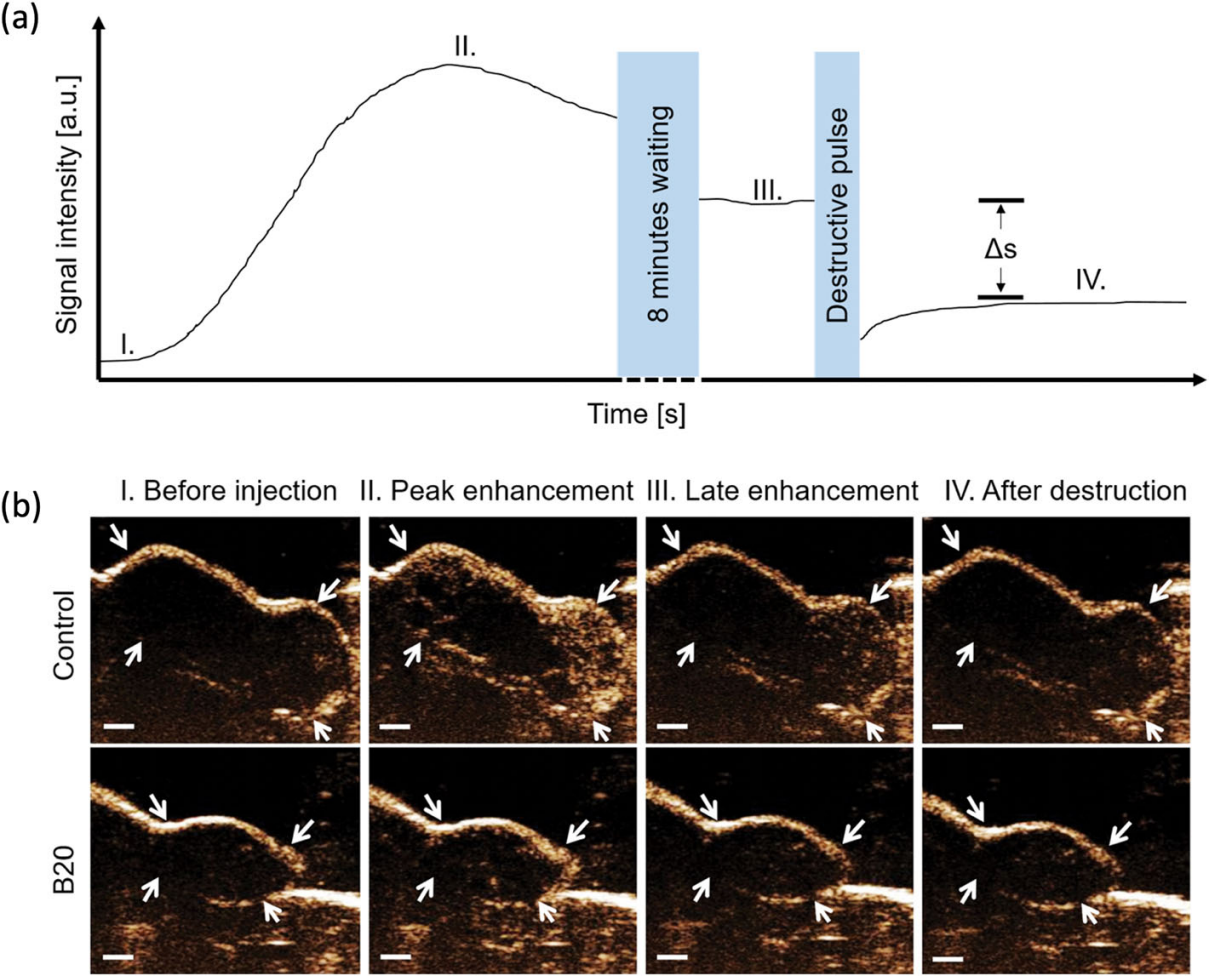
Use of VEGFR2 targeted microbubbles to assess perfusion and angiogenic target expression within the same ultrasound examination. (**a**) Schematic presentation of a typical time-intensity curve using targeted microbubbles. The numbers refer to the respective time points of the images shown in (**b**). (**b**) The upper row shows US images of untreated experimental breast cancers in the contrast mode, the bottom row tumours treated with anti-VEGF antibodies (B20). The microbubble accumulation in the wash-in phase is clearly reduced in treated tumours, indicating a breakdown of vascular function. Consequently, there is reduced binding of VEGFR2 targeted microbubbles in the treated tumours. Destruction of the microbubbles by US confirms that they were stationary at the target (right images) (image adapted from [168])

*Molecular ultrasound imaging (MOUS)* has been shown to detect therapy responses earlier than normal ceUS during anti-angiogenic therapies (Fig. 18) [168]. Clinical studies using BR55, microbubbles targeting the endothelial biomarker vascular endothelial growth factor receptor 2 (VEGFR2), have shown their safety and usefulness for cancer detection in ovarian, breast and prostate lesions [169, 170]. Clinical studies are currently initiated to identify whether and how this information about VEGFR2 can improve clinical decision making in primary diagnosis, cancer characterization, therapy selection and monitoring.

#### Hybrid US imaging (HYBUS)

Combined, or hybrid imaging typically relies on hardware combinations of complementary imaging methods. To date, US has not entered the hybrid imaging field beyond pilot field developments. Tavitian and his team did propose a triple-modality imaging device, by adding ultrafast ultrasound to a PET/CT system, and investigating the energy metabolism and vascular architecture during tumour growth at the same time [171]. In the future, we may see efforts towards integrating US into PET and also MRI systems, so as to overcome the intrinsic spatio-temporal limitations of these modalities through ultrasound-based motion correction during image reconstruction [172]. US-guided motion compensation of PET data may be explored in the future for tracking more complex, non-respiratory motions as well.

#### High-intensity focused ultrasound (HIFU)

The future of HIFU is likely to be MR-guided, so as to leverage high spatial resolution and soft tissue contrast of MR images to delineate a target lesion and its surrounding normal anatomic structures, and to use MR for real-time thermal dose control and treatment monitoring. The use of HIFU can be combined with drug delivery triggered by US-induced hyperthermia or mechanical effects, which can further help in reducing potential tumour relapse from the ablation site [173].

#### Sonoporation and sonopermeabilisation

When microbubbles enter the ultrasound field, they begin to oscillate (stable cavitation). If the amplitude exceeds a certain threshold, the microbubbles undergo inertial cavitation, that is they vigorously oscillate and eventually collapse, thereby generating high speed air jets, which can permeate cell membranes. Both, stable and inertial cavitation, can be used to overcome biological barriers. Sonoporation describes US applications with the intent to improve the transport of drugs or genes through pores generated in cell membranes, while sonopermeabilisation gears towards overcoming vascular and stromal barriers, such as the blood brain barrier (BBB) or matrix fiber networks in desmoplastic tumours. Carpentier et al. showed in a phase I study that repeated and transient BBB opening can be achieved and is well tolerated [174]. In another clinical study, inoperable pancreatic cancer patients were treated successfully with gemcitabine in combination with microbubbles and ultrasound [175]. Despite these promising results, extensive characterization, optimization and safety assessments are required to identify the best setup and settings for its purpose while preventing unwanted and irreversible injury of the tissue. Especially in the treatment of very stromal tumours, as well as for drug delivery across the BBB, sonoporation may improve therapy efficacy and outcome in the future.

**In summary**, the diagnostic and therapeutic potential of US will be explored through translation of early pre-clinical developments. US offers microvascular characterization at almost microscopic level in humans and provides important functional and molecular characteristics that can be used for tissue characterization. In this context, the use of computer-assisted lesion detection and automated image analysis will decrease user-dependency of the modality. Radiomics analysis of ultrasound data will further increase the accuracy and diagnostic impact [176]. In addition, new therapeutic applications of US, e.g., to ablate tissue or to improve drug delivery are emerging. In conjunction with other imaging modalities, US can, thus, be expected to play a significant role in theranostic imaging in the future.

## Optical imaging and instrumentation trends

Optical imaging instrumentation encompasses a large variety of technologies ranging from aided visual inspection using magnifying glasses or video endoscopes, to light microscopy, as well as techniques that require computational efforts to generate diagnostically relevant information. Two thirds of the medical imaging market are defined by optical instrumentations with a mainstay in ophthalmology and endoscopy [177]. With its unique spatial resolution capabilities, optical imaging can be used to assess subcellular structures as well as mesoscopic details of tissue and organs.

### Status quo

Given its limited imaging depth, optical imaging is used mainly to study easily accessible outer surfaces of organs, such as the skin or the inner lumen of organs that can be reached by endoscopes or catheters, such as cardiovascular system, the gastrointestinal tract, oral cavity, larynx down to the lung, bladder and urethra, cervix and uterus. The deepest non-invasive penetration is achieved through the eye down to the retina, thereby making use of the natural transparency of the ocular media. Optical coherence tomography (OCT) is applied with great success in the eye as well as through endoscopes in inner organs [178]. Deeper penetration in tissues can be achieved by diffuse optical spectroscopy and tomography (ODT), which has been successfully combined with non-optical imaging technologies, such as CT, or photo- or optoacoustic imaging (PAI) [179]. Further, Cherenkov luminescence imaging (CLI) is currently being explored for deep optical imaging [180, 181]. In case of invasive intra-surgical application, optical technologies can guide precise tumour excision and help to spare vessels to avoid excessive bleeding. Fluorescence markers are used to enhance image contrast in optical imaging, so as to support tumour detection and image-guided interventions during bioptic sampling and surgical excision.

Light microscopy is the workhorse of standard histopathologic tissue assessment. However, the entire procedure, from tissue sampling to a stained histology, is complex and time consuming. Timelines are critical, in particular during surgical intervention, when tumour borders need to be separated from healthy surrounding tissues. Even when using faster cryo-fixation of biopsies, the examination is still done on stained samples by a pathologist outside the surgical room, which makes it impossible to assess the full tumour margin. This results in inadequate tumour resections with reported 20-70% of cases in breast cancer surgery, or 85% of cases in head and neck surgery [182]. Additional surgery may be required, thus, impairing the quality of life of these patients.

#### Contrast agents

The identification of early-stage cancer is supported by optical imaging technologies. For example, fluorescent markers, such as indocyanine green (ICG), 5-Aminolevulinic acid (5-ALA) [183] or hexyl-aminolevulinate (HAL - Hexvix®) [184] help to delineate tumour boundaries and highlight suspicious tissue regions that go undetected with standard white light illumination. Additional biopsy analysis is still required that benefits from guidance by narrow band imaging (NBI) [185], or optical coherence tomography (OCT).

**Fig. 19:**
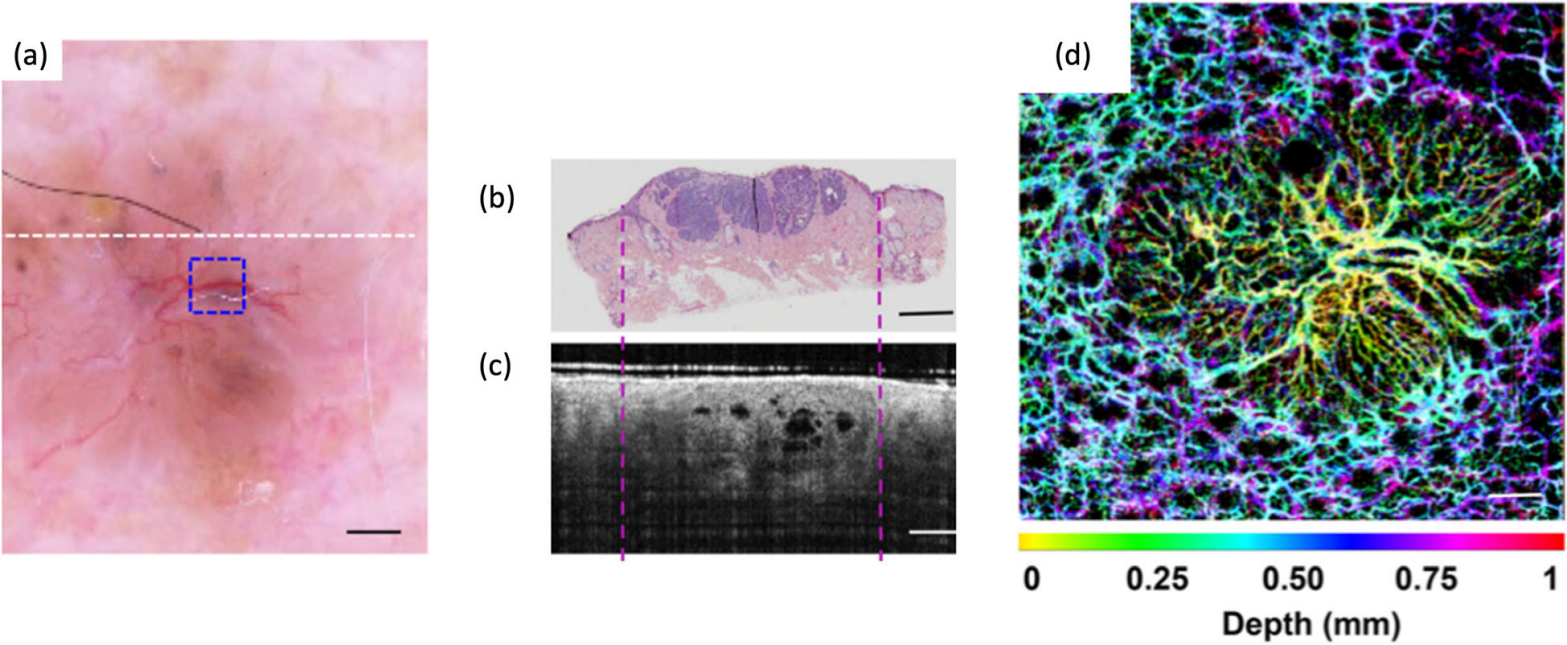
OCT and OCTA of basal cell carcinoma. Scale bar: 1 mm. (**a**) Standard white light dermatoscopy image of the lesion; (**b**). H&E stained biopsy taken at the dotted line in (**a**) indicating the lesion as dark purple area; (**c**) optical coherence tomography (OCT) scan across the lesion along the dotted line in (**a**); (**d**) OCT angiography (OCTA) with colour depth coding for the depth range between 0.1 and 0.5 mm below the skin surface exhibiting a characteristic pattern of neovascular growth to the tumour and with large vessel close to the surface. (reproduced with permission from Wiley and Sons from [189])

**Fig. 20:**
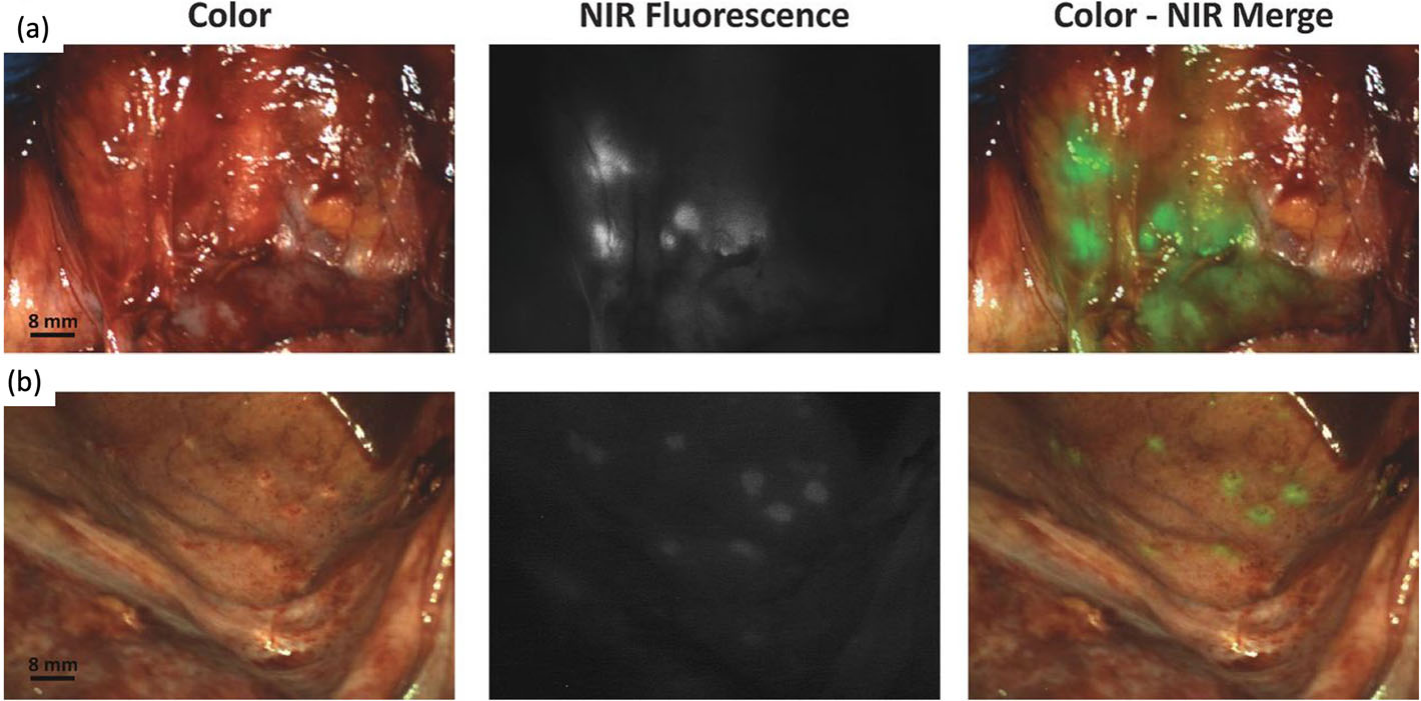
Fluorescence Medical Imaging (FMI) of ovarian cancer metastases: images of retroperitoneal lymph nodes containing metastases of ovarian cancer (**a**) and superficial peritoneal metastases of ovarian cancer (**b**); Left column: white light images revealing low contrast for the lesion areas; middle column: NIR fluorescence images by FMI; right column: fused white light and FMI images for increased contrast of focal mestastases. (reproduced from [191] with permission from AACR Publications)

NBI is limited in penetration depths to 100 μm, or less, thus, visualizing only superficial vessels. OCT, on the other hand, exhibits better penetration into tissue (down to 2 mm) depending on the centre wavelength and tissue type. OCT, and its functional extension OCT angiography, allows to determine the cancer stage by assessing the actual invasiveness of the tumour growth, which is an important parameter to affect treatment decision [186–188] (Fig. 19). Fluorescence Medical Imaging (FMI) has proven effective in various scenarios of intra-surgical guidance. ICG selectively accumulates in tumour cells, presumably due to the enhanced permeability and retention (EPR) effect [190]. Over the last years, FMI or PDD have become important diagnostic tools for brain tumour resection and for the detection and delineation of bladder cancer. Both methods have helped improve surgical treatment of patients with cancers of the breast, ovaries and cervix (Fig. 20). Recent studies suggest that surgeons are able to resect cancerous lesions smaller than a millimeter in size using FMI guidance [192, 193].

#### Resolution versus penetration depth

While optical imaging methods exhibit high spatial resolution, their penetration depth is limited by dominant scattering and absorption effects. Scatter decreases with wavelengths in the visible range, while for longer wavelengths water absorption becomes the dominant effect. The absorption coefficient depends on molecular dynamics and exhibits local maxima (resonances) and minima. In general, medical optical windows are centred at local minima of the water absorption curve at 800 nm, 1060 nm, or 1300 nm.

*Diffuse optical spectroscopy* (DOS) is a well-established medical imaging technology, that operates in these optical windows with reduced scattering and water absorption. It extracts absorption and scattering spectra from deep within tissue associated with concentrations of endogenous and exogenous contrast agents [194]. For example, changes in oxygen saturation indicating the balance between oxy- and deoxyhemoglobine, can be used as a sensitive biomarker of tissue pathologies and for monitoring the effects of therapy [195, 196]. However, the advantages of DOS come at the expense of low spatial resolution.

*Optical coherence tomography* (OCT) acts as a link between high resolution and low penetration cellular microscopy on the one hand and full organ and body imaging with low spatial resolution on the other hand. Today, it has become a reference standard in ophthalmic diagnostics, with a growing field of applications also in other medical disciplines [178]. OCT is capable of producing label-free angiographic maps in 3D, called OCT angiography (OCTA), which has been quickly translated to diagnostic imaging in ophthalmology and dermatology [188, 189, 197].

**In summary**, optical imaging has a long tradition in medical diagnostics, given its unprecedented spatial resolution. Apart from histopathology, optics is widely used in endoscopy, dermatology, and ophthalmology. The most recent optical technique that was successfully translated to the clinics is OCT and OCTA Both provide cross-sectional images of tissue and vasculature with high spatial resolution and with millimeter deep penetration butwith limited tissue specificity. Fluorescence medical imaging significantly improves surgical treatment, thus, resulting in increased life expectation of patients.

### Status go

Currently there are many exciting developments of medical optical technologies in oncology that aim at improving diagnostic specificity, treatment guidance and surgical guidance.

#### Deep tissue imaging

**Fig. 21:**
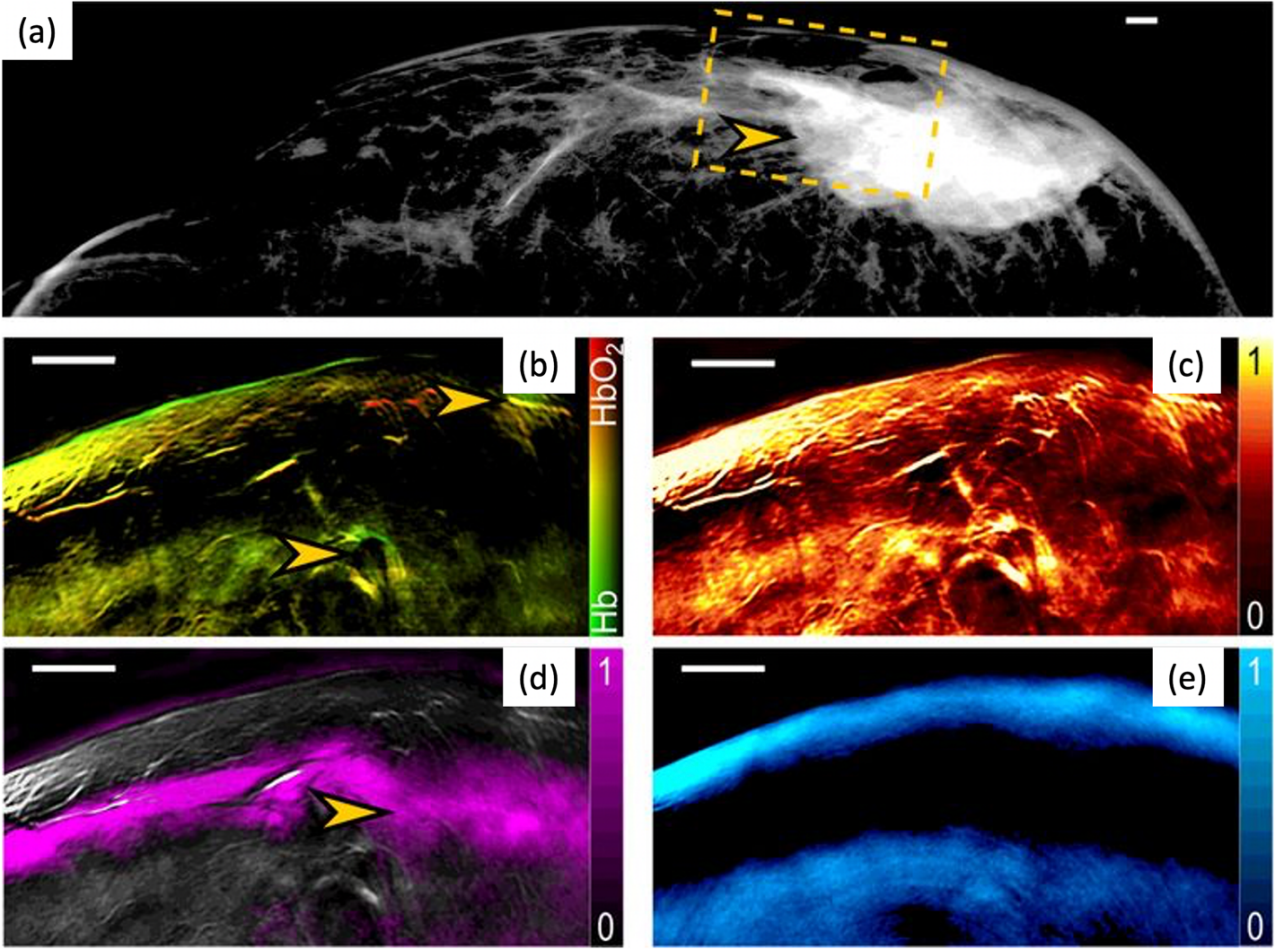
Multispectral Optoacoustics Tomography (MSOT) Imaging of breast cancer: (**a**) X-ray mammography image reveals a  > 4 cm, subcutaneous, non-specific breast tumour; the yellow box indicates the field-of-view of MSOT ; (**b**) MSOT image of Hb and HbO_2_ obtained from the box in (**a**); increased blood volume is indicated by the yellow arrows; (**c**) Total blood volume assessed by MSOT is indicative of hyperemia in the tumour area; (**d**) Relative lipid concentration assessed by MSOT demonstrates disruption of fat layer in the tumour area (arrow); (**e**) relative water concentration assessed by (MSOT). (reproduced from [200] with permission from AACR Publications)

Optical imaging techniques are mostly confined to superficial lesions due to physical limitations of light propagation. Opto- or photoacoustics (PAI), that is the optical excitation of an ultrasound wave in tissue, allows for deeper tissue penetration (about 1cm) [179, 198]. This method uses absorbing endogenous chromophores in tissue, such as hemoglobin or melanin, but could potentially also target exogenous contrast agents. When a short ns-pulse of light is absorbed, it causes local heating and thermo-elastic expansion. This leads to a mechanical pressure wave at high frequency, that propagates to the tissue surface, where it gets detected by an ultrasound sensor. In a recent multi-centre trial with over 2000 women with breast cancer, the specificity of combined PAI and US exceeded that of US alone by 15% with a sensitivity of close to 99%. Advancements in PAI mandate alternative light sources, such as LEDs, that support fast pulse sequences for higher imaging speed. Similar to US, PAI would be have a hand-held probe head for clinical application, calling for real-time or quasi real-time imaging to identify anatomically and pathologically relevant structures [199]. A key strength of PAI is the ability to sense targeted fluorophores deep inside tissues, in addition to visualizing vascular structures and providing oxygen saturation (Fig. 21). Nonetheless, an in-vivo imaging depth of several cm is still a challenge for PAI.

**Fig. 22:**
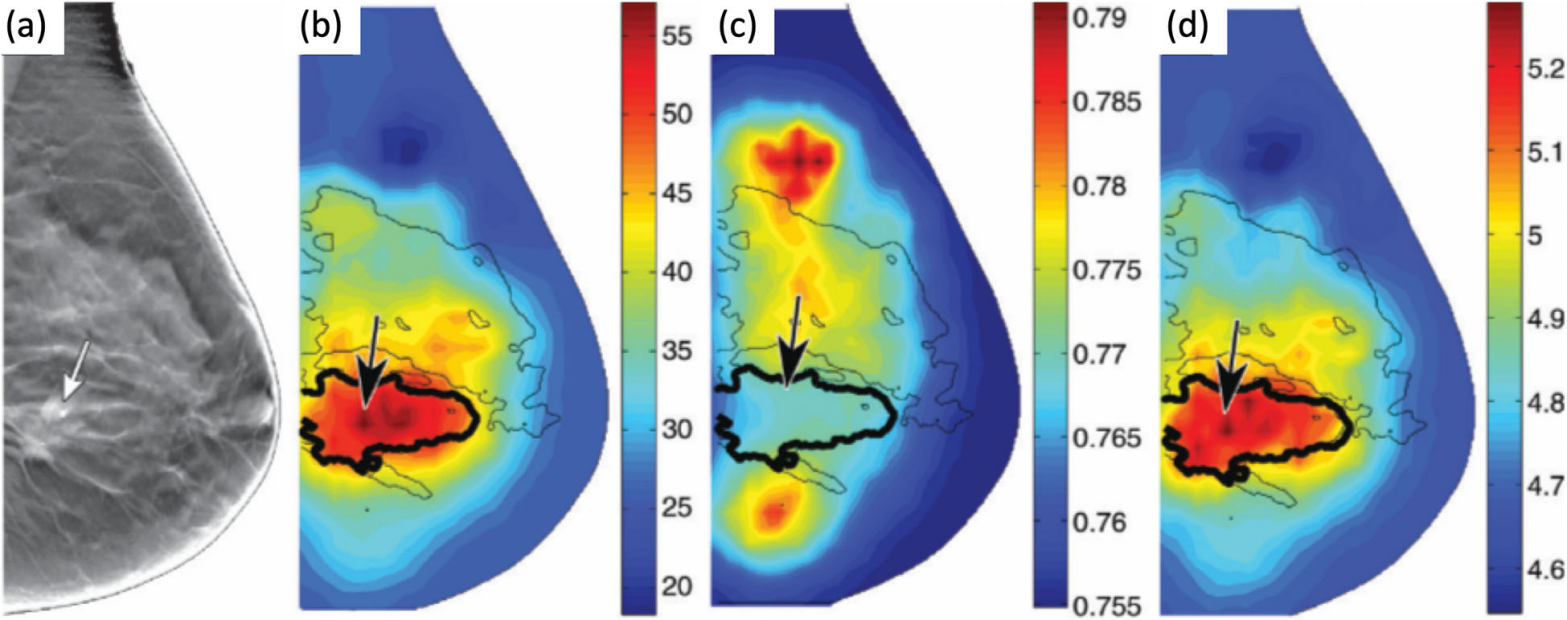
(**a**) Combined X-ray based digital breast tomosynthesis (DBT) and diffuse optical tomography (DOT) of breast cancer: DBT image; (**b**) ODT of total hemoglobin concentration (Hb_T_) in micromoles per liter; (**c**) ODT of oxygen saturation (SO_2_), ODT of reduced scattering coefficient (μ_S_) in cm^− 1^ at a wavelength of 830 nm. (**d**) The breast contains a 2.5-cm invasive ductal carcinoma (arrow on (**a**), black line with arrow on (**b**-**c**). (reproduced from [202] with permission from the Radiological Society of North America)

*Optical Diffuse Tomography (ODT)* shares with ODS the advantage of deep tissue sensing, and overcomes the spatial resolution challenge by using multiple illumination and detection directions. Tomographic reconstruction is based on solving the complex photon diffusion equation incorporating several assumptions on tissue geometry and its optical properties [201]. Nonetheless, in most medical applications the detection space is single-sided and reconstruction is an ill-conditioned problem. The addition of complementary structural imaging yields the required information to locate optical sources in depth (Fig. 22).

*Fluorescence medical tomography (FMT)* is a variant of ODT providing the depth distribution of fluorophores in tissue. The method becomes particularly powerful in combination with targeted exogenous fluorophores emitting in the near infrared region [203].

*Cerenkov luminescence imaging (CLI)* [180, 181] is another emerging medical imaging modality. The radiation is a side effect of radioactive decay, due to particles travelling faster than the speed of light in the respective dielectric medium. Already, there is a variety of PET tracers available that have been approved by national authorities, thus, giving CLI a potential jump start concerning its clinical translation. Nonetheless, the main drawback is its inherently low intensity, and strong scattering and absorption at the typical violet to blue spectral region [204].

#### Multi-modality imaging

A new trend in optical imaging is to replace invasive, and often erratic removal of tissue during biopsy by optical biopsy, i.e., the pathologic assessment of tissue in-situ. Through the combination of high structural sensitivity with molecular sensitivity tumour invasiveness and tumour grading come within reach of multi-modality optical imaging. Given its excellent spatial resolution, OCT comes with a high structural sensitivity while exhibiting low specificity. The latter could be complemented by fluorescence imaging, or by infrared or Raman spectroscopy [205, 206]. Raman spectroscopy has an excellent molecular specificity that results from excitation of rotational and vibrational modes of molecular spectra. The Stokes-shifted emitted light spectrum can be regarded as fingerprint of the assessed substance and exhibits typical regions and resonances for lipids, proteins, etc. Raman scattering is, however, a very ineffective process with less than one photon in a million carrying valuable molecular information. Combining OCT with Raman techniques ex-vivo has been shown to provide insights into urological tumour stages (invasiveness) and grades [186].

Tumour grade is strongly related to its metabolic activity. Metabolites, such as Flavin adenine dinucleotide (FAD) or Nicotinamide adenine dinucleotide (NADH and the redox partner NAD+) have been proven valuable endogenous molecular probes of cell metabolism. Recent developments in fluorescence lifetime imaging (FLIM) show significant changes of FAD and NADH lifetime in tumour versus healthy tissue [207, 208]. Fluorophores are excited in the short visible wavelength range where scattering limits large penetration depths. Multiphoton microscopy, on the other hand, would permit deeper tissue penetration, since the excitation happens at double or triple the wavelength of single photon fluorescence at the cost of non-linear optical processes needing spatio-temporally confined beams with high numerical aperture. The latter calls for close proximity to the tissue, which during intraoperative application can only be achieved through additional hand-held scopes. Moreover, the light sources are bulky and expensive, which limits an easy clinical translation despite promising results in dermatology [209].

#### Targeted exogenous contrast agents

Numerous studies have demonstrated the effectiveness of fluorophore labelling of tumour lesions for enhancing the outcome of tumour resection in different applications. Novel targeted molecular contrast agents are, therefore, extensively studied for precision medicine and are awaiting approval for clinical translation [210, 211]. Hybrid contrast agents are developed supporting modalities with different and often complementary physical contrast mechanisms. Microbubbles for example, that are used in ultrasound imaging, can be modified to incorporate fluorescent agents or radionuclides in their lipid shells supporting US-optical PET imaging. For combining optical imaging and MRI, fluorescent quantum dots with different magnetic properties can be designed to have paramagnetic coatings, or to exhibit high native relaxivity. The contrast agents can further incorporate radionuclide tags to support PET and SPECT imaging. Such multi-modality contrast agents would, for example, allow studying their pharmacokinetics on different scales across various imaging modalities, starting from cell cultures, to preclinical imaging, and finally to humans.

#### Compact systems

Optical imaging technologies are attractive due to their immediate and most intuitive access to tissue and its pathologic changes. They are easily integrated into hand-held devices or hand-held applicators, which is ideal for point-of-care use, as well as for combination with other established imaging technologies. Point-of-care, hand-held endoscopes for cervix screening have been developed and successfully demonstrated by the group of Ramanujam, in particular addressing global health disparities in cancer prevention and disease management [212]. Finally, photonic integrated circuits (PIC) are capable to realize complex optical setups, including sensors, modulators, as well as light sources on a single chip of the size of a cent coin, thus, rendering them ideally suited for compact devices. In the future, wearable devices based on such compact chips may help monitor health status of subjects and as such become integral to disease prevention.

**In summary**, there are many exciting advances of optical technologies to precisely target structure, invasiveness as well as the metabolic activity of tumour cells, so as to replace invasive tissue biopsy extraction. Furthermore, targeting fluorophores or other contrast agents support precision medicine approaches. Navigating regulatory hurdles is key to a faster translation of those novel molecular probes. Optical techniques are on the way to reach deeper into tissue without employing ionizing radiation or as in the case of CLI are a natural add-on to established medical imaging technologies. Non-invasive optical methods may be easily used for large scale screening or for longitudinal treatment monitoring. Future systems provide flexible resolution from organ level down to cellular, make use of the richness of information that light carries targeting specific tissue and cells, being compact, mobile, and are easily combined with other imaging modalities.

## A critical perspective

From a wider perspective, evolution of medical imaging is driven by attempts of making examinations faster, more accurate and, ideally, easier and cheaper to use. Requirements and successes differ for individual modalities. While not exhaustive by design, this section of the paper will attempt to list a number of contenders for improving the quality and applications of medical imaging for cancer patient management (Table 1).

### Modalities

The current differentiation between morphological and functional modalities (Fig. 1) will remain, but the border between them will likely become blurred. Standard morphological modalities will also provide physiological and functional information through more advanced imaging protocols and exploitation of their physical principles through advanced instrumentation and novel contrast agents. Traditional functional modalities will also provide improved morphological cues through new “smarter” instrumentation that not only better exploits the physical properties of detector materials and design, but also circumvents current limitations through novel geometry and processing schemes.

For PET, new total-body designs with enlarged axial FOVs have confirmed a much-increased sensitivity, and low spatial resolution has now become the weakest attribute. New detector designs and detailed modelling of the detector and gantry geometries (incl. Depth-of-interaction, non-collinearity), as well as improved partial volume correction, will markedly increase the spatial resolution up to the physical limits of the modalities. While a recently launched challenge aims at direct imaging through 10 ps TOF PET, further improvements in the meantime will come from image reconstruction techniques (e.g., ML/AI-enhanced) that will take the whole “system” into consideration, that is the subject being imaged, the probe being used in given protocols, and the gantry and detector design as well as all necessary corrections. Another major aspect of PET (and SPECT) are the molecular probes (aka radiotracers) being used for imaging and characterization, with development and validation processes that should be significantly sped up through more advanced in-silico approaches towards first-in-human trials, approval, and clinical deployment. An interesting application, also referred to as “theranostics”, of SPECT and more recently PET, is the use of similar tracers for imaging and therapy, with either different doses or isotopes for each application. In this paradigm, a low radioactive dose scan, either with the same or a different radioisotope, acts as a surrogate that can then be used either directly or through modelling to estimate the internal dose depositions when the actual therapeutic agent is delivered. Such applications will likely further develop alongside targeted personalised molecular therapies that are expected to be much more specific and comprehensive than current surgery, or radiotherapy.

For SPECT, there are many similarities with PET. Larger imaging geometries, this time in the form of ring detectors, will significantly improve volume sensitivity, while the replacement of scintillation detectors with semi-conductor detectors, and/or the replacement of photomultiplier tubes with semiconductor light readout technology will bring significant improvements in energy and spatial resolution. Progress with collimator design together with developments in image reconstruction algorithms will bring further improvements to image quality. These technological advancements will drive the development of theranostics and image-based dosimetry which will ultimately widen the appeal and availability of quantitative SPECT.

For CT, spectral or multi-energy CT has already shown its potential for identifying different materials in the FOV and for removing artefacts, which can be further enhanced through ML/AI approaches. Photon-counting spectral CT has the added benefit of further reducing the ionizing radiation dose from the current lows already afforded by advanced reconstruction techniques. It will also provide much higher spatial resolution, thus, further improving the potential of texture analysis in an advanced analysis regimen. Since spectral CT can also provide information that is relevant to the PET and SPECT energy spectra, it has the potential to produce much more accurate attenuation maps in hybrid modalities (also by unambiguously identifying iodine contrast agent), thus, leading to much improved corrections and reconstructions. Naturally, this potential can be reaped only with the availability of a combined PET/CT offering with spectral CT included, which will depend heavily on market needs. In that case, we anticipate that new contrast agents will also be designed at fast rate that will provide means to qualify physiological processes in complement to more functional data.

For MR, the onus will remain on producing images with better contrast, higher SNR, shorter scan time (translating into more sequences being included in a given examination time for expanded multi-parametric imaging), improved quantification, and higher spatial resolution. For various reasons, including a still very low installed base, the potential of high (up to 7 T) and ultra-high (up from 7 T) fields in the clinic is still unclear, but should the inherent technical limitations (e.g., homogeneity and artefacts) be successfully addressed, such machines will likely be very helpful for moving beyond the current limitations of lower fields by boosting speed, intrinsic SNR and imaging sensitivity (e.g., for fMRI and especially resting state fMRI), while enabling morphological imaging at sub-millimetre spatial resolution. As with CT, new contrast agents will be designed to characterize physiological processes. A special case thereof, which is still not broadly implemented, is hyper-polarized MR by which intrinsic magnetization is enhanced by over four orders of magnitude through dynamic nuclear polarization (DNP), so as to enable real-time monitoring of cell metabolic activities, such as increased pyruvate to lactate conversion in cancer (Warburg effect) [213].

For combined, or hybrid modalities (PET/CT, SPECT/CT and PET/MR) the expected improvements on each of their modality components would potentially yield better corrections (e.g., spectral CT for attenuation and artefact removal) and soft tissue differentiation (e.g., spectral CT again, and faster MR, which will permit more sequences within an acceptable examination period). Combined with more detailed modelling of the imaging instruments, physics and physiology, this will lead to significantly improved image reconstruction and more meaningful imaging and derived information. Further, axial FOVs of PET/CT systems are prone to expand in view of the perceived benefits. Likewise, SPECT/CT may experience a push towards fast and quantitative whole-body imaging, thanks to recent evidence in clinical pilots. While the technical advancement of fully-integrated PET/MR systems is hampered by costs and - still - suboptimal workflow and throughput for a busy clinic, alterative MR-based combinations, such as SPECT/MR [214], may move into the focus of attention, despite similar cost-efficacy concerns. Optical imaging, in combination with other, complimentary imaging modalities, or alone will impact clinical routine in oncology only if the penetration depth challenge can be addressed adequately for probing deep lesions.

Ultrasound has traditionally been seen as highly user-dependent and with low reproducibility, but new 3D transducers will make it more like other tomographic modalities [215, 216]. This will be combined with software that will guide the operator to ensure that all relevant space is scanned and stitched together so that the corresponding volume can be sliced through and analyzed in the most optimal orientation, with real-time performance to build upon the interactive nature of this modality. Contrast agents (e.g., microbubbles) will be further used to provide some functional information, and elastography - ideally through its less user-dependent shear-wave implementation - should also become more prevalent to assess suspected lesions. When combined with ultrafast and super-resolution imaging, it will open further perspectives to microvascular imaging. While ultrafast US imaging requires more advanced transducers and systems, super-resolution imaging based on carefully validated motion models could be applicable to existing machines. More applications can be envisaged through directionally increasing US energy either directly or through nanotech resonators will lead to further applications, such as for heating tissues, making them more prone to taking up drugs and genes via sonoporation, or simply for releasing drugs from nanotech capsules at targeted location.

Optical imaging provides targeted molecular contrast and resolution that is unmatched by other modalities, and, thus, holds great promises for targeted precision medicine even though it is still limited for imaging deep tissue. While this limitation has been successfully addressed through PAI, BLI or CLI in preclinical and research settings, its translation and reproducibility are still lagging. Optical imaging can also relatively easily be combined with other modalities, such as CT, MR or PET, although this again has mostly been demonstrated pre-clinically thus far. A successful implementation of optical imaging is in surgical microscopes, with FMI soon expected to radically improve the efficacy of cytoreductive surgery. While FMI systems are currently limited in specificity due to the paucity of approved fluorophores, FLIM may soon leverage such limitation with several high specificity agents already in the approval pipeline. Perhaps due to a relative ubiquity and low cost of adding optical imaging to other modalities, several multi-modality contrast agents have already been developed for combinations such as Optical/PET, Optical/SPECT, Optical/MR and Optical/US, but their translation to the clinic is still pending. As another special case currently under clinical evaluation, endoscopic OCT provides exquisite differentiation of cancer tissues, also at early and pre-cancerous stages, which can be combined with additional molecular information and Raman spectroscopy for a complete pathologic evaluation.

Photo-acoustic imaging, which uses a laser actuation and US readout, is a modality that has recently emerged and combines the pros (and some cons) of both optical and US imaging to provide an anato-functional insight into live tissues. With its potential already clearly demonstrated in pre-clinical and research settings, this new modality is expected to translate to the clinic soon in combination with specific contrast agents for further expanding its scope. As with US or optical imaging, it will likely be combined with other modalities to complement and expand upon respective findings.

### Quantification

In light of the complexities of cancerous disease, qualitative assessment of images appears not sufficient anymore for detailed and actionable characterisation of extent and staging, or for advanced therapy response assessment. Accordingly, modern evaluation approaches may increasingly rely on extracting or deriving quantitative parameters and information from images, also depending on their inherent type and the modality they are stemming from. Such quantitative biomarker information may provide additional insight into the stage of a disease for a given patient (e.g., lymphoma), where existing consensus criteria have been shown to work well already, or it may soon surpass and replace purely image-based diagnosis, particularly when parametric imaging information is shown to increase specificity of tracer accumulation by better separating cancerous from in-active or inflammatory tissues.

Not all modalities provide equal access on quantitative information in general, or to similar measurements and parameters, which is why it is often key for a disease as complex as cancer to consider them in combination rather than on their own. For morphological modalities, physical measurements can relate to the estimated size (e.g., RECIST or WHO criteria [217, 218], in addition to volume), to the shape of a given lesion (e.g., tortuosity, sphericity, spiculation, etc. [219, 220]), or to structural characteristics as interpreted through imaging (e.g., stereology, BMD, HU in CT; DWI, ADC, DTI in MR; elastography in MR and US; histogram and “texture” in all modalities), all of which require the accurate segmentation of the structures of interest. For functional modalities or protocols, physiological, metabolic and functional characteristics can be extracted, such as respiratory and cardiac motion, dynamic uptake of CT, MR and radioactive tracers or binding of US contrast agents. In the case of PET, individualised metabolic surrogates, such as semi-quantitative SUV [221, 222] or PERCIST [223], can be used, but a truly quantitative approach, such as bona-fide pharmaco-kinetic modelling, which is now re-emerging in clinical settings, will be preferred as it provides much more refined information about the actual inner workings or response of the tissues of interest [224]. Here, leveraging increased volume sensitivities from extended axial FOV coverage will be of the essence for robust quantification even in early-phase, low-count imaging situations shortly after tracer injection, or during follow-up imaging after several half-lives of the injected radioisotopes. Of special note about RECIST and similar metrics that have been primarily developed for/through large clinical trials, while related progression criteria can thus be considered as relevant to and reasonably validated for large cohorts, they may not necessarily be as meaningful for individual patients and pathologies or consistently applied everywhere, and there are still ongoing debates about their optimal use [225–229].

Naturally, standardized imaging pipelines are required to ensure that any differences in measured quantitative value are due to a real functional or morphological change rather than a variation in the imaging process itself; this is irrespective of origin of biomarkers that can be extracted from either morphological modalities or functional modalities. Examples of this standardization process do not only include adherence to high standards of quality control to ensure a repeatable and accurate imaging system performance, but also requires a consistent patient management workflow and robust image analysis tools to extract quantitative biomarker information. In multi-centre trials, this process further extends to harmonizing differences between imaging platforms while frequently relying on contract research organizations (CROs) to standardize the imaging protocols and data analyses.

The recently formulated term of Radiomics now encompasses most extraction - and often combination - of features that initially evolved organically for every modality and are mathematically derived from the digital images at hand [230]. Even before the term Radiomics was coined, the underlying assumption for extracting such features was that, either on their own or in combination with other data, they might be suitable surrogates for underlying biological, physiological or morphological characteristics of particular relevance. Because such approaches tend to be very sensitive to the initial data selection process, some of the main limits to their clinical validation and deployment lies in the variability of source data (e.g., across centres or vendors) and the complexity of identifying regions-of-interest in a robust, consistent and accurate manner. With the advent of better ways to harmonise and extract data, and also of more advanced segmentation techniques informed by statistical atlases and ML techniques, these approaches should become easier to use and more reliable, thus, opening the door to proper validation and eventually broad clinical implementation. At any rate, the main aim of quantitative approaches is to develop and identify robust imaging biomarkers that can be further combined into so-called signatures/fingerprints for diagnosing and characterizing disease as early as possible, and ideally without requiring invasive tissue sampling via biopsies or resections. These biomarkers will then be used for dynamically tailoring therapy regimen and assessing response. However, combining various parameters into a single signature may hide individual contributions, and complex tentative Radiomics signatures should always be carefully assessed [231], with negative results also being of relevance [232].

### Artificial intelligence

Because of the high-dimensionality of multi-modality imaging data – either the source images or the parametrically derived information – and of their cross-disciplinary diversity, their meaningful and actionable analysis and interpretation will increasingly rely on the assistance of computers and advanced algorithms. Accordingly, Machine Learning (ML) and associated Artificial Intelligence (AI) approaches are becoming increasingly ubiquitous in the medical imaging domain, from instrument design and characterization, to acquisition, corrections [233–235] and reconstructions.

While the potential of such approaches has already been demonstrated in various scenarios [236], one should still remain cautious about how the underlying models were trained, and especially whether the data used at the training stage might have introduced some bias into the models [237, 238]. To minimize such biases in either population, pathology or even interpretation, proper ML/AI training tends to require a high number of highly diverse (and high-quality) data in order to cover all possibilities the algorithm may have to deal with, but such numbers and diversity are rarely achievable in practice for medical imaging, and especially so for rarer complex cases that may thus be of most interest for these approaches. As a way to mitigate this inherent limitation, synthetic data can be created from existing ones in order to expand virtually the learning space, but, again, some bias might be introduced by how the simulation model is configured. Another more versatile mitigation strategy, therefore, is to ensure that any results obtained through ML/AI will be fully explainable and come with a confidence score for a human operator to assess.

The ultimate goal is personalized precision medicine that is individually tailored to the patient and condition at hand, as determined by in-vivo biomarkers derived from imaging in combination with other information (e.g., genomics, circulating tumour cells, etc.). While therapies will be applied to real patients, in-vivo imaging and derived information will be key to informing a virtual – in-silico – model of the patient and their pathologies down to the molecular level, on which a virtual therapy regimen can then be devised and refined prior to its real-life deployment.

### Visualization

With ever more high-dimensional data and advanced analysis thereof comes the requirement to develop visualization paradigms able to convey the most significant information to human operators in a clear and unambiguous fashion. Through ad-hoc data reduction techniques and highly interactive systems incorporating multi-dimensional input devices, this can be achieved through standard means such as traditional 2D and 3D displays. The increasing numbers of modalities and complexity of information, as derived from both imaging and non-imaging biomarkers and data [5], to be merged together for a meaningful and actionable representation may still eventually require other approaches, likely derived from other areas, such as games or virtual reality, for more advanced visualization and interaction paradigms also relying on various senses and actuators (e.g., haptics, sound, eye tracking, brain-computer interfaces, etc.). A special case is for planning and guidance during interventional or surgical procedures, where augmented and virtual reality approaches will need to be implemented as un-obstructively as possible to assist with both navigation and delivery. Such approaches will also benefit from/to robotic devices, from mere assistants to semi-autonomous machines.

### Need for training

With new, more capable and complex machines and approaches comes the need to train users so that they can make the most of the added potential and also ensure that expanded capabilities are used with a full understanding of their strengths and limitations [239]. New medical school curriculuma already include some material pertaining to advanced techniques and technology, and conversion degrees (i.e., programmes providing formal training in a separate discipline than one’s core degree) will likely come up to complement the main medical courses with imaging (incl. Modalities and advanced visualization), ML/AI, software engineering, medical physics, etc. similarly to how disciplines, such are biostatistics are currently taught to medical practitioners (physicians, radiographer, technologists, physicists, etc.) who want/need to expand their grasp of disciplines relevant to their clinical work or research interests [240].

## Conclusions

Non-invasive imaging is an integral part of patient management, particularly in oncology. A range of imaging modalities provides a wealth of information encompassing anatomical, functional and molecular data. Imaging modalities are also combined for so-called hybrid imaging concepts if the combination is made of complementary imaging methods and if, as in many cases, such hybrid imaging systems are both feasible and cost effective. Together, technologies and methodologies advance quickly thanks to innovations by clinical researchers and users as well as equipment manufacturers. General trends in these advances include attempts to make imaging faster, more accurate and more amenable to patients. Lately, the use of ancillary machine learning approaches and artificial intelligence has become the focus of attention so as to reduce image distortions, reduce patient exposure or examination time, and last but not least, to assist the clinical readers with supplementary decision support systems. In order to keep pace with the systemic advances of imaging, clinical users and readers are required to continuously update their knowledge and understanding. Finally, non-invasive imaging does provide only a snapshot of the patient, while other biomarkers, including omics, may add to the breadth of diagnostic information. Therefore, a closer integration of imaging and non-imaging methods is expected over the years to come as one of the key components of truly personalized medicine.

## Supplementary information

**Additional file 1: Table S1.** A list of the key performance characteristics of the different detector material that are currently used commercially, NaI is used as a reference scintillator. **Table S2.** Important parameters of state-of-the-art premium CT systems today, including the way how the systems realize dual energy CT. Table adopted with permission from reference [241]. **Figure S1.** Consensus perspective of the co-authors on the use of the key imaging modalities reviewed here for the different stages of cancer patient work-up. Here, *diagnosis* is the image-led process of identifying cancer. *Staging* is the image-supported process of assessing the extent of the disease, incl. Metastatic spread. *Restaging* is the image-led attempt to find out the amount or spread of cancer in the body as the disease returns or intensifies after treatment. Restaging may also be done to find out how the cancer responded to treatment. *Follow-up* describes the image-supported monitoring process of a person’s health over time after treatment. For example, CT imaging is used extensively across all four pillars of cancer patient management while Optical Imaging (OI) plays a significant role primarily during diagnosis and follow-up. **Figure S2.** Key challenges for PET imaging relate to image quality (partial volume effects, image data / noise, randoms, scatter, motion, etc). These challenges were mentioned first in the late 1980s (centre bars, [16–23]). Since then, multiple technological and methodological advances have been made that help address these challenges. TX = Transmission, recon = image reconstruction. Here, the thickness of the connectors describes the magnitude of the cross-correlation.

## Data Availability

NA

## Abbreviations

ADC: Apparent diffusion coefficients
AI: Artificial intelligence
AMD: Age-related macular degeneration
BBB: Blood brain barrier
BCC: Basal cell carcinoma
CE: Contrast-enhanced
CERT: Cerenkov radiance energy transfer
CEST: Chemical exchange saturation transfer
ceUS: contrast enhanced US
CHESS: Chemical shift selective saturation
CLI: Cherenkov luminescence imaging
CSF: Cerebral spinal fluid
CT: Computed Tomography
CT-AC: CT-based attenuation correction
CZT: Cadmium Zinc Telluride
DECT: Dual-energy CT
DDG: Data-driven gating
DNP: Dynamic nuclear polarization
DOI: Depth of interaction
DOS: Diffuse optical spectroscopy
DTI: Diffusion tensor imaging
DWI: Diffusion weighted imaging
EPR: Enhanced permeability and retention
FAD: Flavin adenine dinucleotide
FBP: Filtered back projection
FLIM: Fluorescence lifetime imaging
FMI: Fluorescence Medical Imaging
FOV: Field of view
GDP: Gross domestic product
HIFU: High intensity focused ultrasound
HU: Hounsfield unit
HYBUS: Hybrid ultrasound
ICG: Indocyanine green
IR: Iterative reconstruction
LNT: Linear no threshold
MAR: Metal artifact correction
ML: Machine learning
MLAA: Maximum likelihood attenuation and activity
MOUS: Molecular ultrasound imaging
MRA: MR angiography
MRI: Magnetic Resonance Imaging
NADH: Nicotinamide adenine dinucleotide
UL: Motion model Ultrasound Localization Microscopy
NBI: Narrow band imaging
NMR: Nuclear Magnetic Resonance
NMAR: Normalized metal artifact reduction
OCT: Optical Coherence Tomography
OCTA: OCT angiography
ODT: Diffuse optical spectroscopy and tomography
OSEM: Ordered subset expectation maximization
PAI: Photo- or optoacoustic imaging
PC: Phase-contrast
PET: Positron Emission Tomography
PIC: Photonic integrated circuits
PMT: Photomultiplier tube
PVE: Partial volume effect
REFI: Radiopharmaceutical-excited fluorescence imaging
RF: Radiofrequency
SAR: Specific absorption rate
SCIFI: Secondary Cerenkov-induced fluorescence imaging
SPECT: Single Photon Emission Computed Tomography
SiPM: Silicon PMT
SMS: Simultaneous multi-slice
SNR: Signal to noise ratio
SUV: Standardized uptake value
TOF: Time of flight
ULM: Ultrasound Localization Microscopy
US: Ultrasound
USE: Ultrasound elastography
ZTE: Zero time echo
5-ALA: 5- Aminolevulinic acid
