# Supplementary material for: What scans we will read: imaging instrumentation trends in clinical oncology
Source: Cancer Imaging. 2020 Jun 9;20:38. doi: 10.1186/s40644-020-00312-3 (PMC7285725; doi:10.1186/s40644-020-00312-3)
Supplement: Supplementary file 1 — Additional file 1: Table S1. A list of the key performance characteristics of the different detector material that are currently used commercially, NaI is used as a reference scintillator. Table S2. Important parameters of state-of-the-art premium CT systems today, including the way how the systems realize dual energy CT. Table adopted with permission from reference [241]. Figure S1. Consensus perspective of the co-authors on the use of the key imaging modalities reviewed here for the different stages of cancer patient work-up. Here, diagnosis is the image-led process of identifying cancer. Staging is the image-supported process of assessing the extent of the disease, incl. Metastatic spread. Restaging is the image-led attempt to find out the amount or spread of cancer in the body as the disease returns or intensifies after treatment. Restaging may also be done to find out how the cancer responded to treatment. Follow-up describes the image-supported monitoring process of a person’s health over time after treatment. For example, CT imaging is used extensively across all four pillars of cancer patient management while Optical Imaging (OI) plays a significant role primarily during diagnosis and follow-up. Figure S2. Key challenges for PET imaging relate to image quality (partial volume effects, image data / noise, randoms, scatter, motion, etc). These challenges were mentioned first in the late 1980s (centre bars, [16–23]). Since then, multiple technological and methodological advances have been made that help address these challenges. TX = Transmission, recon = image reconstruction. Here, the thickness of the connectors describes the magnitude of the cross-correlation. [file 40644_2020_312_MOESM1_ESM.docx]

**Suppl. table 1** A list of the key performance characteristics of the different detector material that are currently used commercially, NaI is used as a reference scintillator.


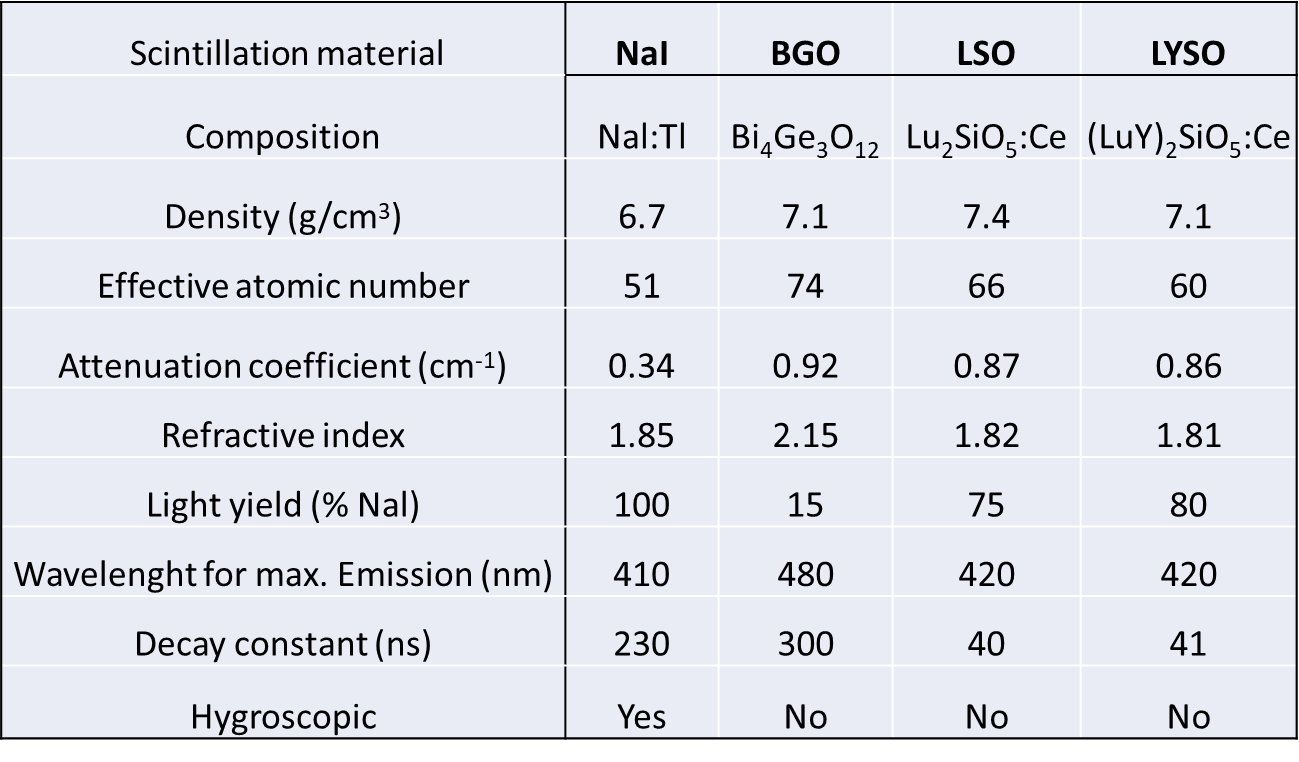


**Suppl. table 2.** Important parameters of state-of-the-art premium CT systems today, including the way how the systems realize dual energy CT. Table adopted with permission from reference [236].


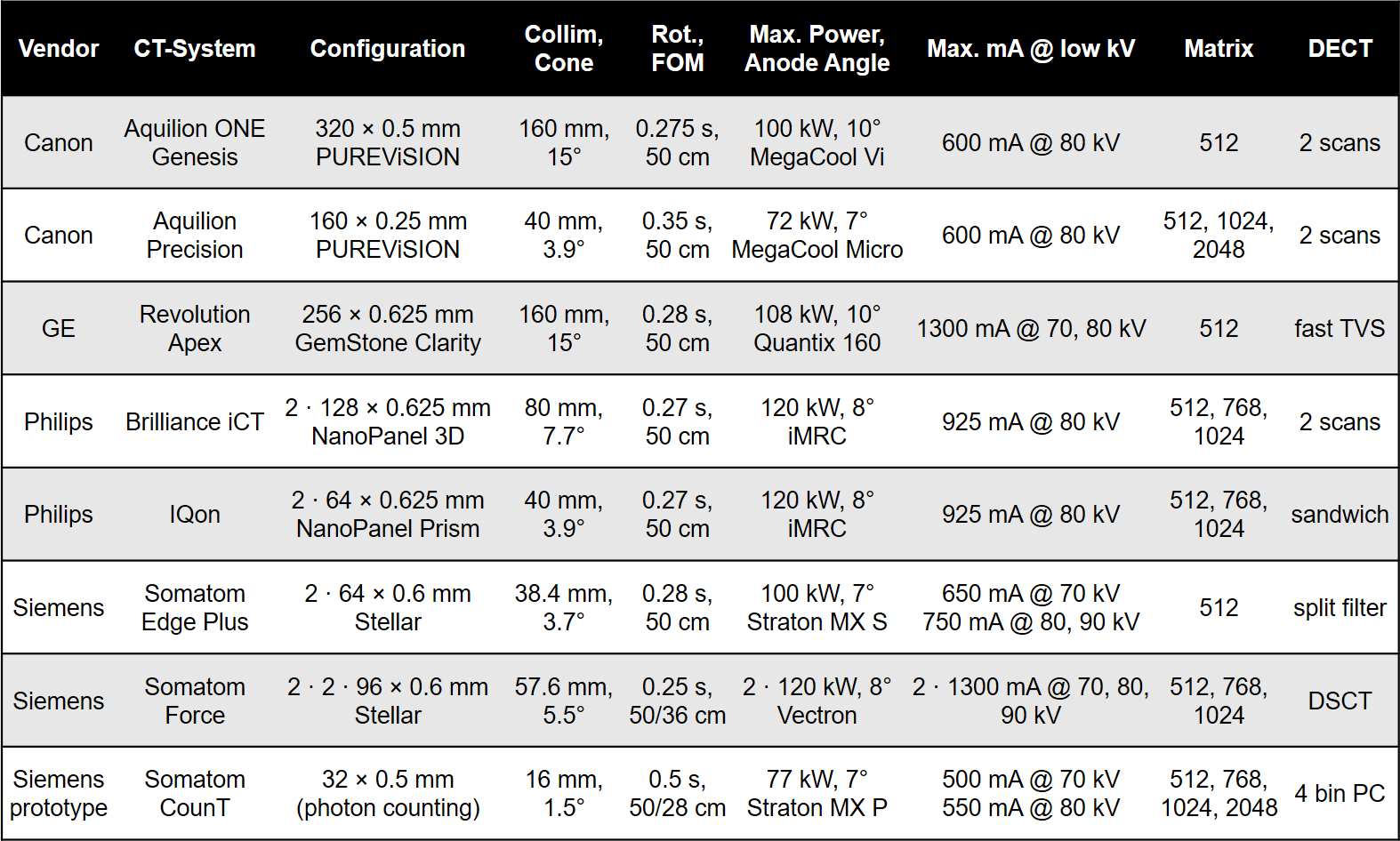


**
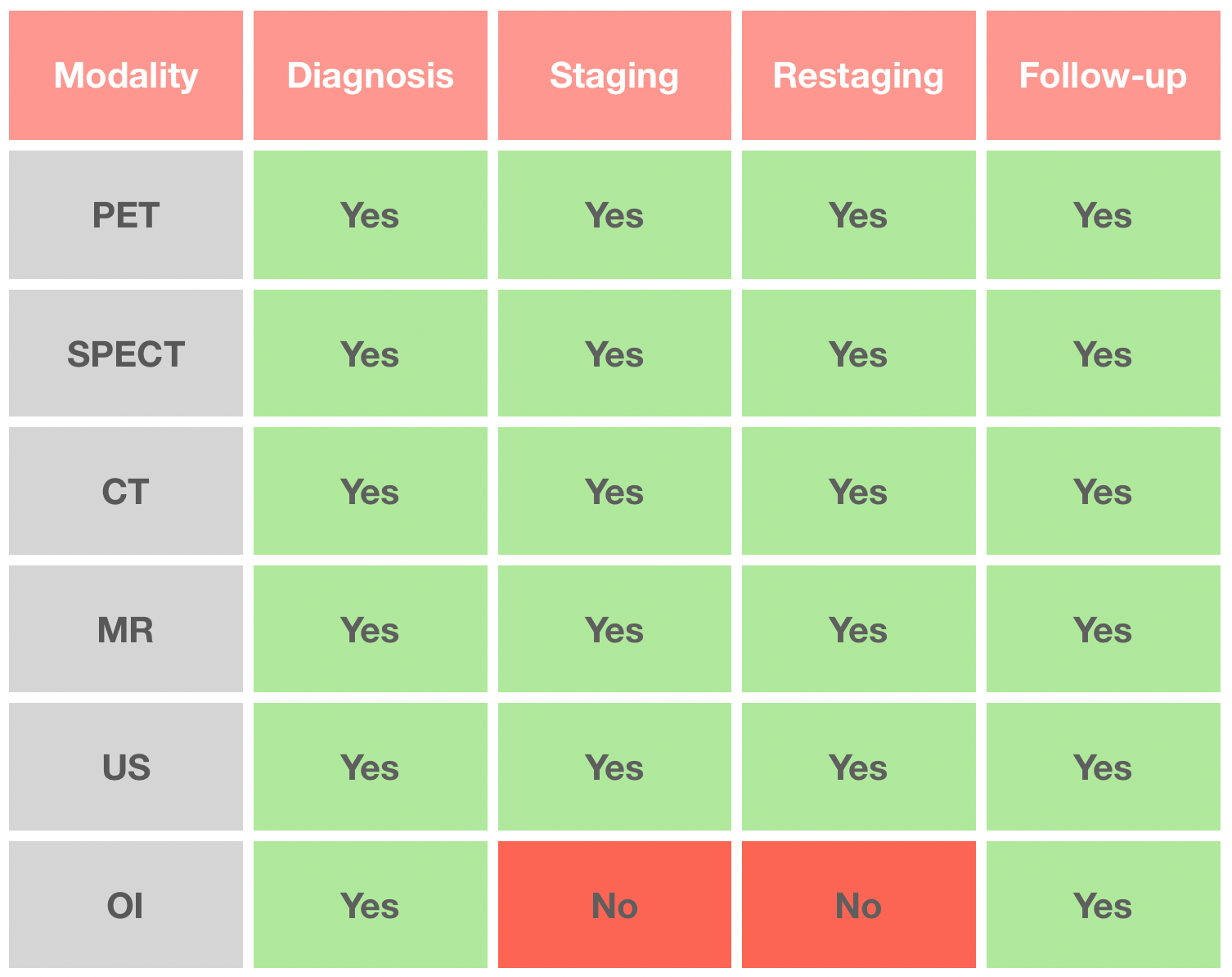
**

**Suppl. Figure 1.** Consensus perspective of the co-authors on the use of the key imaging modalities reviewed here for the different stages of cancer patient work-up. Here, *diagnosis* is the image-led process of identifying cancer. *Staging* is the image-supported process of assessing the extent of the disease, incl. Metastatic spread. *Restaging* is the image-led attempt to find out the amount or spread of cancer in the body as the disease returns or intensifies after treatment. Restaging may also be done to find out how the cancer responded to treatment. *Follow-up* describes the image-supported monitoring process of a person's health over time after treatment. For example, CT imaging is used extensively across all four pillars of cancer patient management while Optical Imaging (OI) plays a significant role primarily during diagnosis and follow-up.


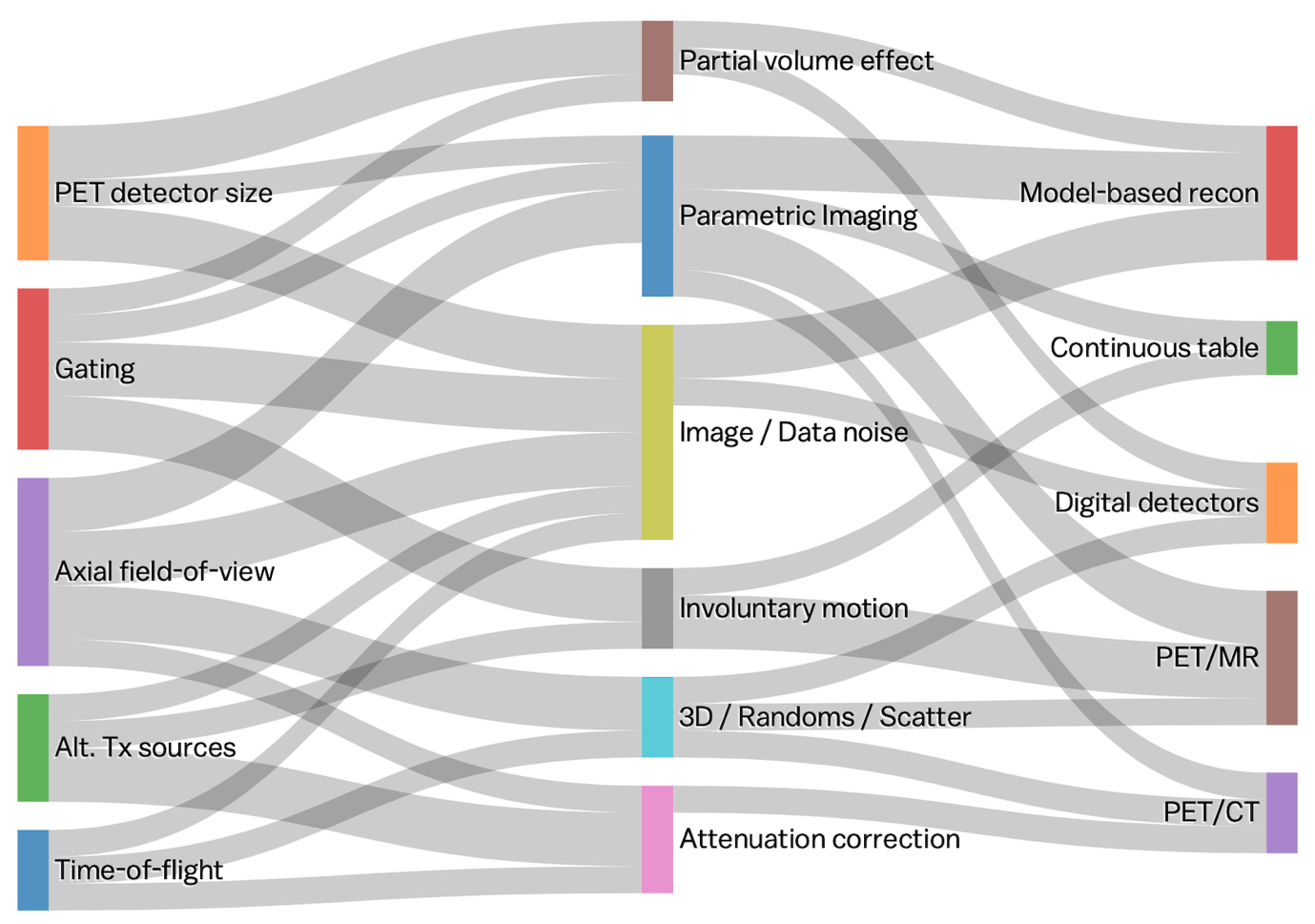


**Suppl Figure 2.** Key challenges for PET imaging relate to image quality (partial volume effects, image data / noise, randoms, scatter, motion, etc). These challenges were mentioned first in the late 1980s (centre bars, [16–23]). Since then, multiple technological and methodological advances have been made that help address these challenges. TX=Transmission, recon = image reconstruction. Here, the thickness of the connectors describes the magnitude of the cross-correlation.
